# Epigenome-wide association study on asthma and chronic obstructive pulmonary disease overlap reveals aberrant DNA methylations related to clinical phenotypes

**DOI:** 10.1038/s41598-021-83185-1

**Published:** 2021-03-03

**Authors:** Yung-Che Chen, Ying-Huang Tsai, Chin-Chou Wang, Shih-Feng Liu, Ting-Wen Chen, Wen-Feng Fang, Chiu-Ping Lee, Po-Yuan Hsu, Tung-Ying Chao, Chao-Chien Wu, Yu-Feng Wei, Huang-Chih Chang, Chia-Cheng Tsen, Yu-Ping Chang, Meng-Chih Lin, Taiwan Clinical Trial Consortium of Respiratory Disease (TCORE) group, Chong-Jen Yu, Hao-Chien Wang, Chi-Huei Chiang, Diahn-Warng Perng, Shih-Lung Cheng, Jeng-Yuan Hsu, Wu-Huei Hsu, Tzuen-Ren Hsiue, Hen-I. Lin, Cheng-Yi Wang, Yeun-Chung Chang, Chung-Ming Chen, Cing-Syong Lin, Likwang Chen, Inn-Wen Chong

**Affiliations:** 1 Division of Pulmonary and Critical Care Medicine, Department of Medicine, Kaohsiung Chang Gung Memorial Hospital and Chang Gung University College of Medicine, Niao-Sung District, 123, Ta-Pei Rd, Kaohsiung, 83301 Taiwan; 2 Medical Department, College of Medicine, Chang Gung University, Taoyuan, Taiwan; 3 Molecular Medicine Research Center, Chang Gung University, Taoyuan, Taiwan; 4 Bioinformatics Center, Chang Gung University, Taoyuan, Taiwan; 5 Institute of Bioinformatics and Systems Biology, National Chiao Tung University, Hsinchu, 30068 Taiwan; 6 Chang Gung University of Science and Technology, Chia-Yi, Taiwan; 7 Department of Respiratory Therapy, Kaohsiung Chang Gung Memorial Hospital and Chang Gung University College of Medicine, Kaohsiung, Taiwan; 8 Department of Internal Medicine, E-Da Hospital, I-Shou University, Kaohsiung, Taiwan; 9 National Taiwan University Hospital, Taipei, Taiwan; 10 Taipei Veterans General Hospital, Taipei, Taiwan; 11 Far Eastern Memorial Hospital, New Taipei City, Taiwan; 12 Taichung Veterans General Hospital, Taichung, Taiwan; 13 China Medical University Hospital, Taichung, Taiwan; 14 National Cheng Kung University Hospital, Tainan, Taiwan; 15 Cartinal Tien Hospital, Taipei, Taiwan; 16 National Taiwan University, Taipei, Taiwan; 17 Changhua Christian Hospital, Changhua, Taiwan; 18 National Health Research Institutes, Miaoli, Taiwan; 19 Kaohsiung Medical University Chung-Ho Memorial Hospital, Kaohsiung, Taiwan

**Keywords:** Genetics, Systems biology, Biomarkers, Diseases, Medical research, Molecular medicine, Pathogenesis

## Abstract

We hypothesized that epigenetics is a link between smoking/allergen exposures and the development of Asthma and chronic obstructive pulmonary disease (ACO). A total of 75 of 228 COPD patients were identified as ACO, which was independently associated with increased exacerbations. Microarray analysis identified 404 differentially methylated loci (DML) in ACO patients, and 6575 DML in those with rapid lung function decline in a discovery cohort. In the validation cohort, ACO patients had hypermethylated *PDE9A* (+ 30,088)/*ZNF323* (− 296), and hypomethylated *SEPT8* (− 47) genes as compared with either pure COPD patients or healthy non-smokers. Hypermethylated TIGIT (− 173) gene and hypomethylated *CYSLTR1* (+ 348)/*CCDC88C* (+ 125,722)/*ADORA2B* (+ 1339) were associated with severe airflow limitation, while hypomethylated IFRD1 (− 515) gene with frequent exacerbation in all the COPD patients. Hypermethylated *ZNF323* (− 296) / *MPV17L* (+ 194) and hypomethylated *PTPRN2* (+ 10,000) genes were associated with rapid lung function decline. In vitro cigarette smoke extract and ovalbumin concurrent exposure resulted in specific DNA methylation changes of the *MPV17L* / *ZNF323* genes, while 5-aza-2′-deoxycytidine treatment reversed promoter hypermethylation-mediated *MPV17L* under-expression accompanied with reduced apoptosis and decreased generation of reactive oxygen species. Aberrant DNA methylations may constitute a determinant for ACO, and provide a biomarker of airflow limitation, exacerbation, and lung function decline.

## Introduction

A large proportion (15–30%) of patients with chronic airways disease has features of both asthma and chronic obstructive pulmonary disease (COPD) (Asthma and COPD Overlap, ACO). ACO patients experience more frequent exacerbations, have poorer quality of life, decline in lung function more rapidly, and consume a disproportionate amount of healthcare resources than asthma or COPD alone^1,2^. Up to date, no universal definition criteria exist^3–7^, and few studies have investigated the pathogenesis of this overlap syndrome^8,9^. Only 25% of life-long smokers develop COPD, and asthma exhibit a strong familial connection, suggesting genetic determinants in susceptibility to both COPD and asthma^10,11^. In the past three decades, extensive research to identify genetic determinants of COPD and asthma has shown that only a few single nucleotide polymorphisms (SNP) are independently and consistently associated with fixed airflow limitation^12,13^. Epigenetics, which refers to the process of influencing gene expression through other genetic mechanisms without affecting DNA sequences, may accounts for this discrepancy.

DNA methylation occurring at position 5 of the pyrimidine ring of cytosines in the context of the cytosine followed by guanine dinucleotide sequence (CpG) form the basis of epigenetic mechanisms through inhibiting the binding of transcription factors at the promoter regions or influencing transcriptional elongation and alternative splicing at the intragenetic regions. Gene promoter methylation often leads to transcriptional repression of the gene, whereas gene body methylation is frequently associated with high gene expression levels^14,15^. DNA methylation patterns are not only inheritable but also susceptible to change in response to environmental stimuli, such as smoking and allergens^16^. Additionally, SNPs in non-coding regions may simultaneously alter both the consensus sequence and its DNA methylation, if they alter or generate CpG dinucleotides^17^. Recent candidate gene and epigenome-wide association studies (EWAS) have identified several CpG site-specific aberrant DNA methylation changes associated with COPD and asthma individually^18–21^, but none has been performed in this overlap group^22^. We hypothesized that gene-specific CpG methylation profiles of peripheral blood mononuclear cells (PBMCs) may contribute to disease susceptibility, severity, and clinical phenotypes in ACO patients, with the goal of identifying novel epigenetic changes related to frequent exacerbation, rapid lung function decline, or severe airflow limitation.

## Results

### Clinical characteristics of the whole cohort

A total of 75 of the 228 COPD patients were identified as ACO, while the others classified as pure COPD (Table 1). Using the new GOLD 2019 staging system (A–D), ACO group (A: 20.8%, B: 38.9%, C: 13.9%, D: 26.4%) had a greater proportion of patients categorized as C and D (40.3% versus 24.8%, p = 0.018) compared with pure COPD group (A: 32.7%, B: 42.5%, C: 7.2%, D: 17.6%). The numbers of all (1.6 ± 2 versus 0.9 ± 1.3, p = 0.013) and moderate (1 ± 1.5 versus 0.5 ± 0.9, p = 0.009) exacerbations in the past one year were higher in ACO group versus pure COPD group. Among 156 patients who received follow-up 1 year later, the numbers of all (1.6 ± 2 versus 0.9 ± 1.3, p = 0.033) and moderate exacerbation (1 ± 1.39 versus 0.6 ± 0.9, p = 0.032) in the next one year were also higher in the ACO group (n = 55) versus the pure COPD group (n = 101). Stepwise forward multivariate linear regression analysis showed that the presence of ACO (co-efficient 0.519, 95% CI 0.06 to 0.518, p = 0.027) and a higher modified Medical Research Council (mMRC) value (co-efficient 0.355, 95% CI 0.169 to 0.541, p < 0.001) were independent factors associated with total number of exacerbations in the past one year, while the presence of ACO (co-efficient 1.212, 95% CI 0.507 to 1.918, p = 0.001), the use of inhaled corticosteroids (ICS) and long-acting β2 agonist (LABA) combination therapy (co-efficient 1.368, 95% CI 0.498 to 2.237, p = 0.003), and a lower post-bronchodilator (BD) forced expiratory volume in one second (FEV1) %predicted value at visit 2 (co-efficient − 0.026, 95% CI − 0.05 to − 0.003, p = 0.027) were independent factors associated with total number of exacerbations in the next one year.

**Table 1:** Baseline characteristic of all study participants with asthma-COPD overlap (ACO) or pure COPD.

	ACO N = 75	Pure COPD, N = 153	P value
Age, years	69.2 (10.6)	68.8 (10)	0.794
Smoking exposure, pack-years	50.5 (31.9)	55.7 (36.7)	0.296
Current smoker	20 (26.7)	76 (49.7)	0.001
Body mass index, Kg/m^2^	24.2 (5.3)	24.2 (4.3)	0.968
Charlson co-morbidity index	2.7 (1.6)	2.5 (1.6)	0.233
**Atopic disease, n (%)**	50 (66.7)	54 (35.3)	< 0.001
Asthma	23 (30.7)	17 (11.1)	< 0.001
Allergic rhinitis	40 (53.3)	50 (32.7)	0.003
Atopic dermatitis	4 (5.5)	5 (3.3)	0.433
**Lung function**			
Pre-BD FEV1/FVC, %	54.2 (10.2)	57.3 (13.8)	0.06
Pre-BD FEV1, %predicted	55 (17.7)	59.1 (19.8)	0.124
Pre-BD FEF25-75%, %predicted	22.7 (11.2)	27.4 (14.6)	0.01
Post-BD FEV1/FVC, %	57.8 (11.1)	58.8 (13.5)	0.588
Post-BD FEV1, %predicted	61.5 (18.4)	62.1 (19.4)	0.821
Post-BD FEF25-75%, %predicted	27.9 (13.1)	29.4 (15.5)	0.473
BD responsive	75 (100)	31 (22)	< 0.001
**Dyspnea score**			
mMRC at the first visit	1.7 (1.1)	1.6 (1.2)	0.422
CAT at the first visit	11.4 (7.8)	10.3 (7.3)	0.322
**Blood and biochemistry test**			
Neutrophil, %	58.1 (11.8)	63.3 (13.3)	0.005
Eosinophil, %	4.5 (4.3)	2.7 (3.5)	0.002
Absolute neutrophil count, μL^−1^	4384 (2048)	5054 (2654)	0.063
Absolute eosinophil count, μL^−1^	312 (273)	213 (492)	0.106
Total cholesterol	181.8 (37.4)	182.5 (40.5)	0.904
Triglyceride	115.8 (60.3)	115.1 (71.1)	0.947
Uric acid	7.6 (2.2)	6.9 (1.7)	0.018
Glycohemoglobin	5.9 (0.5)	6.1 (1)	0.014
**Controller medicines, n (%)**			
LAMA	33 (44)	68 (44.4)	0.949
LABA	34 (45.3)	65 (42.5)	0.683
ICS + LABA	31 (41.3%)	53 (34.6)	0.325
Theophylline	46 (61.3)	69 (45.1)	0.021
**Exercise endurance test**			
Maximum inspiratory pressure, cmH2O	70.1 (30.5)	70.5 (27.1)	0.922
Maximum expiratory pressure, cmH2O	101.9 (40.1)	96.2 (35.9)	0.324
6 min walking distance, m	388.5 (103.1)	368.6 (129.5)	0.29
6 min walking distance, %predicted	81.4 (21.3)	76.9 (25.6)	0.341

*COPD* chronic obstructive pulmonary disease, *BD* bronchodilator, *FEV1* forced expiratory volume within first second, *FVC* forced expiratory vital capacity, *FEF* forced expiratory flow, LAMA = long acting muscarinic antagonist, *LABA* long acting β2 agonist, *ICS* inhaled corticosteroid, *mMRC* modified Medical Research Council, *CAT* COPD assessment test.

### Whole-genome DNA methylation profiles and enrichment pathway analysis in the discovery cohort

**Figure 1:**
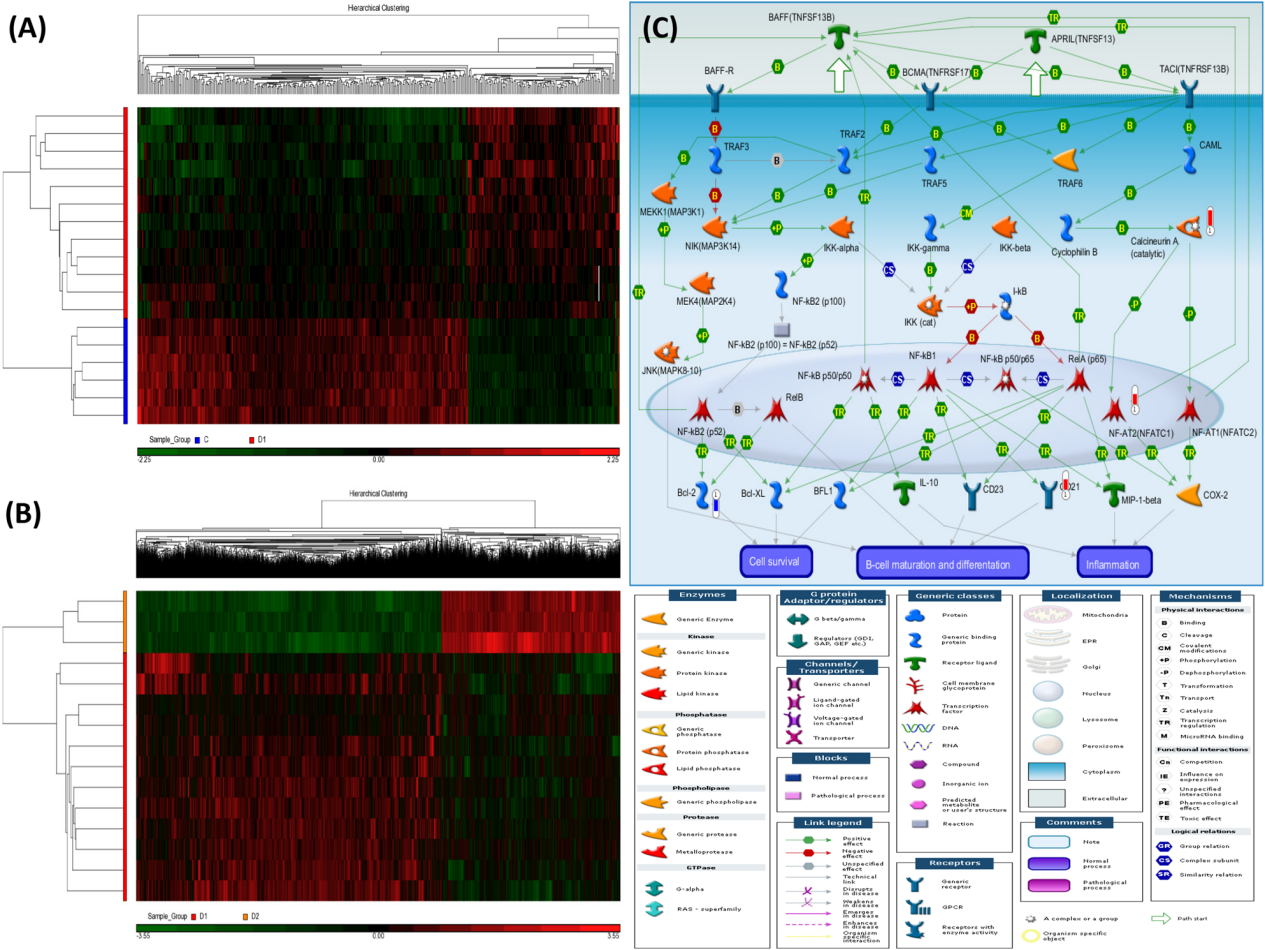
Heatmaps and a representative enriched pathway of the differentially methylated loci (DML) for the two comparisons of the whole genome microarray experiment in the discovery cohort. Hierarchical clustering of DML in 21 samples classified into two comparisons: (**A**) ACO patients versus healthy subjects (comparison I). (**B**) ACO patients with rapid decline in lung function after 1-year follow-up versus at enrollment (comparison II). (**C**) Apoptosis and survival of *APRIL* and *BAFF* signaling pathway enriched in ACO patients (comparison I). The significantly hypermethylated genes were highlighted with a red-colored barometric bar, while hypomethylated genes in a blue-colored bar. The changes represent the differences between the mean β-values of normal and ACO patients. For example, the mean β-value for normal and ACO patients is 1.44 and 1.94, respectively, for *calcineurin A* (*CACNA1C*), indicating a higher methylation level (+ 0.5) in ACO. The image was created by the Metacore software.

Twelve ACO patients and 6 healthy non-smokers (HS) enrolled in the discovery cohort were matched in terms of age, BMI, and Charlson co-morbidity index (Supplementary Table S1). A total of 21 PBMC samples were grouped and analyzed in two different comparisons. The first comparison (I) was between 12 ACO patients and 6 HS, resulting in 125 hypermethylated differentially methylated loci (DMLs) and 279 hypomethylated DMLs (all p values < 0.0005, all q values < 0.3; Fig. 1A, Table 2). The second comparison (II) was before and after 1-year follow-up in 3 ACO patients with rapid lung function decline, resulting in 2432 hypermethylated DMLs and 4143 hypomethylated DMLs (all p values < 0.005, all q values < 0.3; Fig. 1B, Supplementary Table S2). For the 404 DMLs in comparison I, enrichment in previous EWAS signals was tested by using EWAS toolkit (https://bigd.big.ac.cn/ewas/toolkit)^23^. The results showed that there is high co-occurrence probability between the 404 probes and smoking, air pollution, aging, atopy, or autoimmune disease-related DNA methylation probes in previous EWAS signals. Furthermore, 19 DMLs in comparison I overlapped with asthma trait-related DNA methylation probes (Supplementary Table S3), but none overlapped with COPD trait-related probes. *TNRC6B* and *MET* were hypermethylated in both of our ACO patients and the asthma patients in previous EWAS, while *DHX30*, *SFXN*, *C19orf28*, and *CLCN7* were hypomethylated.

**Table 2:** Top differentially methylated loci in the comparison between patients with asthma and COPD overlap (ACO) and healthy non-smokers (comparison I) in the discovery cohort.

Column ID	UCSC RefGene Name	UCSC RefGene Accession	UCSC RefGene Group	P value	q value	Mean difference 0f. β value
cg16093065	*TNRC6B*	NM_001162501	3′UTR	0.000430942	0.174101	0.179
cg07674304	*WWOX*	NM_016373	Body	0.000215471	0.0870503	0.173
cg14150023	*TNRC6B*	NM_001162501	3′UTR	0.000215471	0.0870503	0.159
cg13828808	*ROR2*	NM_004560	Body	0.000430942	0.174101	0.137
cg19421218	*TIGIT*	NM_173799	TSS200	0.000107735	0.0435249	0.132
cg26601310	*PRR5L*	NM_001160167	5′UTR	0.000215471	0.0870503	0.131
cg14392772	*TRAF1*	NM_005658	3′UTR	0.000430942	0.174101	0.127
cg24450112	*PDE9A*	NM_001001582	Body	0.000430942	0.174101	0.119
cg22027471	*SLC5A4*	NM_014227	TSS1500	0.000430942	0.174101	0.115
cg05757530	*NLRC5*	NM_032206	5′UTR	0.000430942	0.174101	0.113
cg07563400	*ADORA2B*	NM_000676	Body	0.000215471	0.0870503	− 0.116
cg17278447	*NPTX2*	NM_002523	Body	0.000430942	0.174101	− 0.116
cg22422264	*USP50*	NM_203494	3′UTR	0.000430942	0.174101	− 0.116
cg13676763	*FAM125B*	NM_033446	Body	0.000430942	0.174101	− 0.118
cg03707168	*PPP1R15A*	NM_014330	Body	0.000430942	0.174101	− 0.119
cg13334727	*SEPT8*	NM_001098813	TSS200	0.000215471	0.0870503	− 0.119
cg14459011	*NHEDC2*	NM_178833	TSS1500	0.000107735	0.0435249	− 0.121
cg20861489	*HCRTR2*	NM_001526	Body	0.000430942	0.174101	− 0.122
cg01534527	*CTDSPL*	NM_005808	Body	0.000215471	0.0870503	− 0.123
cg12655112	*EHD4*	NM_139265	Body	0.000430942	0.174101	− 0.123
cg07568296	*MAD1L1*	NM_003550	Body	0.000430942	0.174101	− 0.123
cg15243578	*PITPNM2*	NM_020845	3′UTR	0.000430942	0.174101	− 0.124
cg08510456	*BSN*	NM_003458	TSS1500	0.000430942	0.174101	− 0.125
cg15545247	*GPR109B;*	NM_006018	1stExon	0.000430942	0.174101	− 0.125
cg02853948	*HCRTR2*	NM_001526	Body	0.000430942	0.174101	− 0.125
cg20705781	*SSH3*	NM_017857	TSS1500	0.000430942	0.174101	− 0.125
cg18200150	*MYO1D*	NM_015194	Body	0.000107735	0.0435249	− 0.126
cg04658021	*PER1*	NM_002616	TSS1500	0.000430942	0.174101	− 0.127
cg01376079	*SSH3*	NM_017857	TSS1500	0.000215471	0.0870503	− 0.127
cg07987148	*TP53RK*	NM_033550	TSS1500	0.000430942	0.174101	− 0.127
cg18688704	*PDGFC*	NM_016205	Body	0.000430942	0.174101	− 0.129
cg07180646	*TMEM51*	NM_001136218	5′UTR	0.000430942	0.174101	− 0.13
cg22331200	*MPO*	NM_000250	Body	0.000430942	0.174101	− 0.134
cg06487194	*JARID2*	NM_004973	Body	0.000430942	0.174101	− 0.135
cg22499893	*SFRS13A*	NM_054016	TSS1500	0.000215471	0.0870503	− 0.135
cg01394781	*ABCC1*	NM_019862	Body	0.000430942	0.174101	− 0.137
cg12401918	*NOTCH4*	NM_022107	TSS1500	0.000430942	0.174101	− 0.137
cg06815976	*NOTCH4*	NM_004557	Body	0.000430942	0.174101	− 0.137
cg15529344	*ANKRD58*	NM_001105576	TSS1500	0.000430942	0.174101	− 0.138
cg26337070	*ATOH8*	NM_032827	Body	0.000430942	0.174101	− 0.143
cg24892069	*NRP1*	NM_001024628	Body	0.000215471	0.0870503	− 0.146
cg02607972	*ASXL2*	NM_018263	3′UTR	0.000430942	0.174101	− 0.147
cg01836137	*INF2;*	NM_022489	5′UTR	0.000430942	0.174101	− 0.152
cg11615395	*MAML3*	NM_018717	Body	0.000107735	0.0435249	− 0.152
cg05655915	*NARF*	NM_012336	TSS1500;	0.000430942	0.174101	− 0.152
cg00813999	*CYSLTR1*	NM_006639	1stExon	0.000430942	0.174101	− 0.153
cg19351604	*ARHGEF10*	NM_014629	Body	0.000430942	0.174101	− 0.154
cg05413628	*CLCN7*	NM_001287	Body	0.000430942	0.174101	− 0.157
cg01870865	*TREX1*	NM_016381	TSS200	0.000215471	0.0870503	− 0.159
cg25918947	*TMEM106A*	NM_145041	Body	0.000430942	0.174101	− 0.16
cg07375836	*ACACA*	NM_198839	5′UTR	0.000430942	0.174101	− 0.165
cg26746309	*ERLIN1*	NM_001100626	Body	0.000215471	0.0870503	− 0.165
cg14481208	*RTKN*	NM_001015055	TSS1500	0.000430942	0.174101	− 0.165
cg09841842	*FRMD6*	NM_001042481	5′UTR	0.000215471	0.0870503	− 0.166
cg08223235	*BCL2*	NM_000633	Body	0.000215471	0.0870503	− 0.167
cg20981848	*BTBD3*	NM_014962	Body	0.000215471	0.0870503	− 0.167
cg10718056	*TRIM27*	NM_006510	Body	0.000430942	0.174101	− 0.167
cg19628988	*CXXC5*	NM_016463	5′UTR	0.000430942	0.174101	− 0.169
cg21141827	*ETF1*	NM_004730	3′UTR	0.000215471	0.0870503	− 0.179
cg17514528	*MTHFR*	NM_005957	Body	0.000215471	0.0870503	− 0.184
cg16672562	*HIF3A*	NM_022462	1^st^ Exon	0.000430942	0.174101	− 0.226
cg10288111	*IFRD1*	NM_001007245	TSS1500	0.000430942	0.174101	− 0.243
cg11307715	*DENND3*	NM_014957	Body	0.000430942	0.174101	− 0.361

*UTR* un-translated region; *TSS* transcription start site.

The top-ranking pathways enriched in comparison I included apoptosis and survival of *APRIL* and *BAFF* signaling (Fig. 1C), immune response of *NF-AT* signaling in leukocyte interactions (Supplementary Fig. S1), and development role of *HDAC* and *CaMK* in control of skeletal myogenesis (Supplementary Table S4). The top-ranking pathways enriched in comparison II included *NF-AT* signaling, regulation of epithelial-to-mesenchymal transition, and *TGF/WNT* signaling for cytoskeletal remodeling (Supplementary Table S5).

### Differential PDE9A, SEPT8, and ZNF323 gene methylations with respect to the presence of ACO in the validation cohort

The 22 ACO patients, 48 pure COPD patients, and 10 HS enrolled in the validation cohort were matched in terms of age, BMI, and Charlson co-morbidity index (Supplementary Table S6).

**Figure 2:**
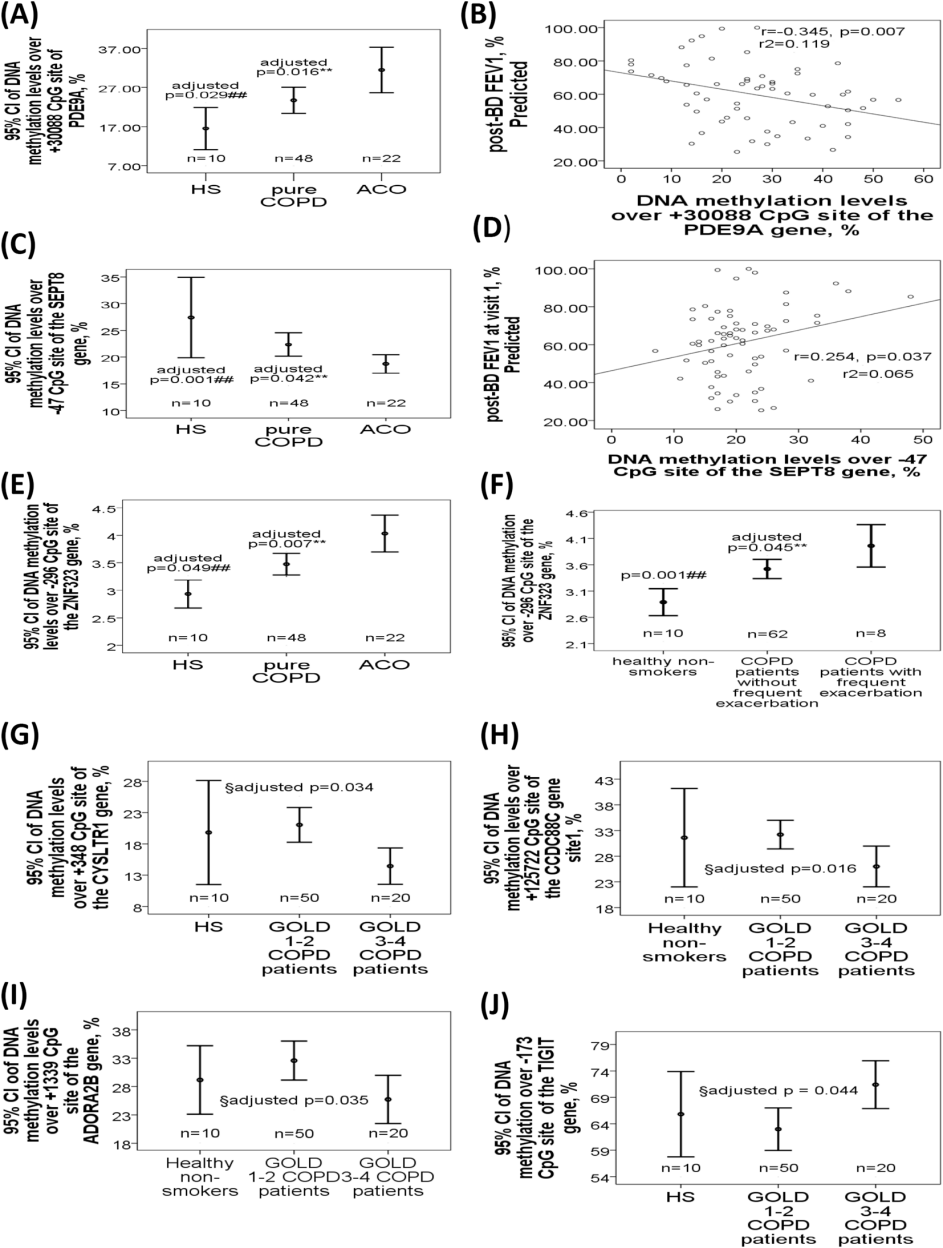
Differential DNA methylation patterns of the *PDE9A*, *SEPT8*, *ZNF323*, *CYSLTR1*, *TIGIT*, *ADORA2B*, and *CCDC88C* genes at cross sectional levels in the validation cohort. (**A**) DNA methylation levels of the *PDE9A* gene body (+ 30,088 CpG site) were increased in ACO patients versus either pure COPD patients or healthy subjects (HS), and (**B**) negatively correlated with post-BD FEV1%predicted. (**C**) DNA methylation levels of the *SEPT8* gene promoter region (− 47 CpG site) were decreased in ACO patients versus either pure COPD patients or HS, and (**D**) positively correlated with post-BD FEV1%predicted. (**E**) DNA methylation levels of the *ZNF323* gene promoter region (− 296 CpG site) were increased in ACO patients versus either pure COPD patients or HS, and (**F**) also increased in all the COPD patients with frequent exacerbation versus those without frequent exacerbation or HS. (**G**) DNA methylation levels of the *CYSLTR1* gene promoter region (+ 348 CpG site) were decreased in GOLD III-IV COPD patients versus GOLD I-II COPD patients. (**H**) DNA methylation levels of the *CCDC88C* gene body (+ 125,722 CpG site) were decreased in GOLD III-IV COPD patients versus GOLD I-II COPD patients. (I) DNA methylation levels of the *ADORA2B* gene body (+ 1339 CpG site) were decreased in GOLD III-IV COPD patients versus GOLD I-II COPD patients. (**J**) DNA methylation levels of the *TIGIT* gene promoter region (− 172 CpG site) were increased in GOLD III-IV COPD patients versus GOLD I-II COPD patients. **Compared between ACO and pure COPD patients, and adjusted by multivariate linear regression. ^##^Compared between ACO and healthy non-smokers (HS), and adjusted by multivariate linear regression. ^§^Compared between COPD patients with GOLD I-II and GOLD III-IV, and adjusted by multivariate linear regression.

*PDE9A* gene (+ 30,088, Fig. 2A) was hypermethylated in ACO patients versus pure COPD patients or HS, and negatively correlated with post-BD FEV1%predicted (Fig. 2B). *SEPT8* gene (− 47, Fig. 2C) was hypomethylated in ACO patients versus pure COPD patients or HS, and positively correlated with post-BD FEV1%predicted (Fig. 2D). *ZNF323* gene (− 296, Fig. 2E) was hypermethylated in ACO patients versus pure COPD patients or HS, and increased in all COPD patients with frequent severe AE versus those without frequent severe AE or HS (Fig. 2F).

**Figure 3:**
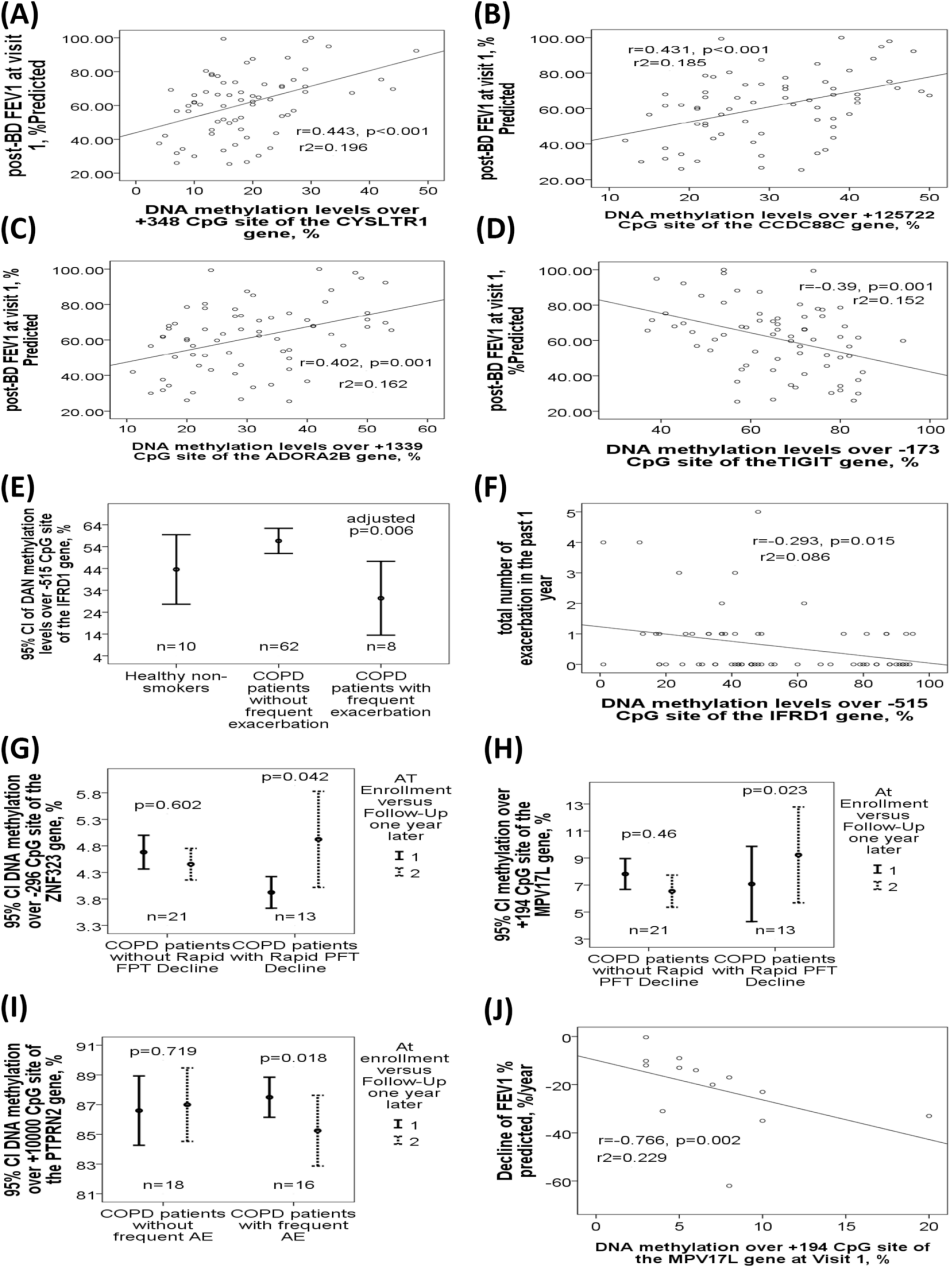
Differential DNA methylation patterns of the *IRFD1*, *TIGIT*, *CysLTR1*, *ADORA2B*, *CCDC88C*, *ZNF323,* and *MPV17L*, and *PTPRN2* genes at cross sectional or longitudinal levels in the validation cohort. (**A**) DNA methylation levels of the *CYSLTR1* gene promoter region (+ 348 CpG site) were positively correlated with post-BD FEV1%predicted value. (**B**) DNA methylation levels of the *CCDC88C* gene body (+ 125,722 CpG site) were positively correlated with post-BD FEV1%predicted value. (**C**) DNA methylation levels of the *ADORA2B* gene body (+ 1339 CpG site) were positively correlated with post-BD FEV1%predicted value. (**D**) DNA methylation levels of the *TIGIT* gene promoter region (− 172 CpG site) were negatively correlated with post-BD FEV1%predicted value. (**E**) DNA methylation levels of the *IFRD1* gene promoter region (− 515 CpG site) were decreased in all the COPD patients with frequent exacerbation versus those without frequent exacerbation, and (**F**) negatively correlated with the number of exacerbations in the past one year. (**G**) DNA methylation levels of the *ZNF323* gene promoter region (− 296 CpG site) were elevated after 1-year follow-up in COPD patients with rapid lung function decline versus that at enrollment. (**H**) DNA methylation levels of the *MPV17L* gene (+ 194 CpG site) were elevated after 1-year follow-up in COPD patients with rapid lung function decline versus that at enrollment. (**I**) DNA methylation levels of the *MPV17L* gene (+ 194 CpG site) at visit 1 were negatively correlated with the difference in FEV1% predicted values between visit 2 and visit 1. (**J**) DNA methylation levels over + 10,015 CpG site of the *PTPRN2* gene were reduced after 1-year follow-up in COPD patients with frequent exacerbation versus that at enrollment.

*CYSLTR1* (+ 348, Fig. 2G), *CCDC88C* (+ 125,722, Fig. 2H), and *ADORA2B* (+ 1339, Fig. 2I) genes were all hypomethylated in COPD patients with severe to very severe airflow limitation (GOLD III-IV) versus those with mild to moderate airflow limitation (GOLD I-II), while *TIGIT* gene (− 173, Fig. 2J) was hypermethylated. *CYSLTR1* (+ 348, Fig. 3A), *CCDC88C* (+ 125,722, Fig. 3B), and *ADORA2B* (+ 1339, Fig. 3C) gene methylations were all positively correlated with post-BD FEV1%predicted, while *TIGIT* gene methylation (− 173, Fig. 3D) was negatively correlated with post-BD FEV1%predicted. *IFRD1* gene methylation (− 515, Fig. 3E) was decreased in all COPD patients with frequent severe AE versus those without frequent severe AE, and negatively correlated with exacerbation frequency (Fig. 3F).

### Differential ZNF323, MPV17L, and PTPRN2 gene methylations with respect to rapid lung function decline in the validation cohort

DNA methylation levels over 7 CpG sites of 7 selected genes from comparison II were measured in 5 ACO patients and 8 pure COPD patients with rapid lung function decline after 1-year follow-up (FEV1%predicted 69.78 ± 12.15 versus 60.72 ± 12.18%, mean difference 9.06 ± 7.54%, p = 0.002), as well as in 12 ACO patients and 9 pure COPD patients without rapid lung function decline.

*ZNF323* (− 296, Fig. 3G) and *MPV17L* gene methylations (+ 194, Fig. 3H) were both elevated after 1-year follow-up (visit 2) versus at enrollment (visit 1) in those with rapid lung function decline, but remained the same in those without rapid lung function decline, while *MPV17L* gene methylation at visit 1 and visit 2 (Fig. 3I) were both negatively correlated with the difference in FEV1% predicted values between visit 2 and visit 1. *PTPRN2* gene methylation (+ 10,000, Fig. 3J) were reduced after 1-year follow-up in 16 patients with frequent moderate to severe AE, and remained the same in those without frequent moderate to severe exacerbation.

### Effects of in vitro concurrent cigarette smoke extract (CSE) and ovalbumin (OVA) stimuli on DNA methylation levels or gene expressions of the 10 candidate genes

**Figure 4:**
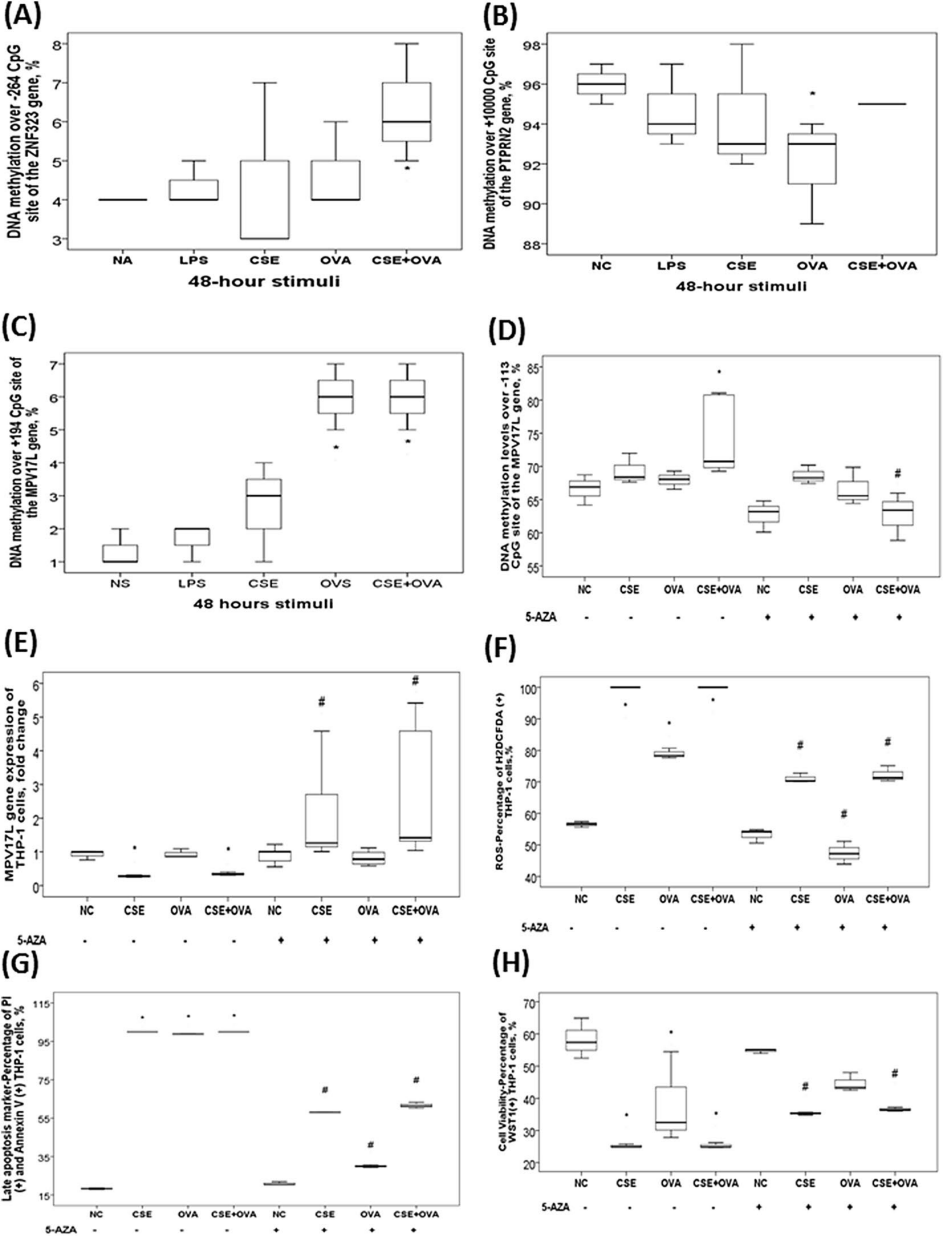
Aberrant DNA methylation and corresponding gene expression changes of the candidate genes in THP-1 cells in response to in vitro cigarette smoke extract (CSE) plus ovalbumin (OVA) allergen stimuli. (**A**) DNA methylation level of the *ZNF323* gene (-264) was increased in response to CSE plus OVA treatment. (**B**) DNA methylation level of the *PTPRN2* gene (+ 10,000) was decreased with OVA stimuli. (**C**) DNA methylation levels over + 194 CpG site of the *MPV17L* gene were increased in response to OVA alone or CSE plus OVA concurrent treatment. Pre-treatment with de-methylation agent (5-AZA) resulted in (**D**) decreased DNA methylation levels over -113 CpG site of the *MPV17L* gene, (**E**) increased *MPV17L* gene expression, (**F**) reduced reactive oxygen species (ROS) production (percentage of H2DCFDA positive cells), (**G**) reduced late apoptosis (percentage of Annexin V and PI double positive cells), and (**H**) increased cell viability (percentage of WST-1 positive cells), as compared with that of CSE, OVA, or CSE plus OVA treatment alone. *p < 0.05 compared between normal control (NC; culture medium) and specific stimuli by Kruskal Wallis H-test. ^#^p < 0.05 compared between the comparative groups with and without 5-AZA supplement by Kruskal Wallis H-test.

*ZNF323* gene methylation (− 264) was increased in response to CSE plus OVA treatment (p < 0.05, Fig. 4A). *PTPRN2* gene methylation (+ 10,000) was decreased with OVA stimuli (p < 0.05, Fig. 4B). *MPV17L* gene (+ 194) methylation was increased in response to CSE plus OVA treatment (p values < 0.05, Fig. 4C). Pre-treatment with de-methylation agent (5-aza-2ʹ-deoxycytidine; 5-aza) in the presence of CSE plus OVA stimuli resulted in decreased methylation over − 113 CpG site of the *MPV17L* gene, increased *MPV17L* gene expression, reduced reactive oxygen species production, reduced late apoptosis, and increased cell viability, as compared with CSE plus OVA treatment alone (all p values < 0.05, Fig. 4D–H), whereas DNA methylation levels of the other 8 candidate genes were not altered despite increased gene expressions (Supplementary Fig. S2).

## Discussion

T helper type 2 immune gene signals associated with greater BD reversibility, eosinophilia, and better response to inhaled corticosteroids, have been identified in ACO patients^8,24^. Although one report is available on sputum DNA methylation changes of the *PCDH20* and *SULF2* genes in relation to ACO, and one genome-wide association study has identified SNPs in the *CSMD1*, *SOX5* and *GPR65* genes for ACO^25,26^, this is the first study to perform a whole genome DNA methylation analysis in ACO with replication of the principal findings, and identify a specific association of ACO and several clinical phenotypes with aberrant DNA methylation patterns.

Aberrant methylation patterns of the *PDE9A*, *SEPT8*, and *ZNF323* genes showed the most significant associations with ACO. PDE9A is the most selective for cGMP degradation, and its inhibitor can enhance memory function through promoting synaptic plasticity and counter pathological remodeling of the heart^27–30^. Given that PDE-4 is responsible for metabolizing adenosine 3′,5′-cyclic monophosphate that reduces the activation of a wide range of inflammatory cells including eosinophils, targeting PDE9A signaling pathway may be a novel strategy for managing ACO^31^. SEPT8 contributes to kidney and liver fibrosis, and modulates the generation of toxic amyloid-beta peptides in Alzheimer's disease^32,33^. Given that SEPT8 functions in various biological processes of cell cytokinesis and migration, it may be another novel target for ACO^34^. Interestingly, using the BiosQTL database (https://genenetwork.nl/biosqtlbrowser/), there is evidence that the effect of the *SEPT8* CpG site (cg13334727) methylation on whole blood may be genetically regulated by the cis-methylation quantitative trait loci (meQTL), rs39855. This SNP is associated with several respiratory phenotypes in the United Kingdom Biobank (UKBB) such as asthma, hay fever or allergic rhinitis, or it is in linkage disequilibrium with the lead variants associated with asthma and respiratory diseases based on the information from the OpenTarget Genetics database (https://genetics.opentargets.org/variant). Our in vitro experiments showed increased gene expressions but no significant changes in the methylation levels for 8 of the 11 selected genes, suggesting that the altered methylation patterns in patients may be inheriting epigenotypes rather than changes after cigarette smoke exposures. Several SNPs of the *ZNF323* (*ZSCAN31*) gene are associated with lung function in asthmatic patients^35,36^, while its hypermethylation was noted both in ACO patients and in response to CSE plus OVA stimuli, suggesting a role of acquired ZNF323 hypermethylation in the pathogenesis of ACO.

Aberrant methylation patterns of the *TIGIT*, *CYSLTR1*, *CCDC88C*, and *ADORA2B* genes were associated with severe airflow limitation. TIGIT can enhance Th2 response^37^, and shows aberrant methylation in allergic asthma^38^. Interestingly, the probe (cg19421218) annotated to *TIGIT* is described as an expression quantitative trait methylation (eQTM) for the same gene in the BiosQTL database. CysLTR1 mediates bronchoconstriction and eosinophil migration in asthma^39,40^. *CCDC88C* genetic mutation is implicated in eosinophilia-associated myeloid/lymphoid neoplasms^41^. ADORA2B contributes to pulmonary fibrosis and pulmonary hypertension associated with COPD^42–44^. Based on the information from the BiosQTL and OpenTarget Genetics databases, DNA methylation of the *ADORA2B* CpG site (cg07563400) may be genetically regulated by two Cis-meQTL, rs3925260 and rs12452624, which have been linked to lung function parameters and asthma, respectively. Our results suggest that these epigenotypes related to allergic responses may be important determinates of lung function in early life and predispose individuals to COPD.

Aberrant methylation patterns of the *MPV17L*, *ZNF323*, and *PTPRN2* genes associated with rapid lung function decline were identified, while altered methylations of the *ZNF323* and *IFRD1* with frequent exacerbation. MPV17L protects against mitochondrial oxidative stress and apoptosis by activation of Omi/HtrA2 protease, and its methylation may act as a biomarker for the prognosis of lung adenocarcinoma^45–48^. Our in vitro experiments showed that de-methylation agent could reverse promoter hypermethylation-mediated under-expression of the *MPV17L* gene and oxidative stress-mediated cell apoptosis in response to concurrent CSE and OVA stimuli, supporting the use of this epigenetic mark as potential therapeutic targets of ACO. Aberrant *PTPRN2* gene methylation has been identified in smoking-related COPD patients in two previous EWAS^49,50^. IFRD1 regulates the pathogenesis of asthma and cystic fibrosis through mediating neutrophil function^51,52^.

There are several limitations in the present study. First, the sample size of the discovery cohort is relatively small and the analyses of both the DMLs and enriched pathways would have not met a more stringent threshold for significance of false discovery rate < 0.5 or 0.1. Three fifths of the results in the comparison I would not remain significant after applying a more stringent threshold of q-value of 0.1 to control the rate of false positives, but all the results in the comparison II remain significant. There would be 162 DMLs with 118 hypomethylated and 44 hypermethylated in the comparison I, if q value is less than 0.1. However, we used a 2-tiered approach to verify some of the findings. Moreover, testing for differentially methylated regions where the cumulative effect of probes included in the same genomic region will be conducted in the future. Second, the cause and effect relationship could not be straightforward determined in this association study. CSE and OVA co-exposure resulted in under-expression of the *MPV17L* gene and hypermethylation of its promoter region, which were in accordance with the findings in the clinical samples and fit the scientific consensus of an anti-correlation between promoter methylation and gene expression. Third, peripheral blood mononuclear cells are comprised of a mixture of cell types, which may contribute to different methylation changes. Single-cell multi-omic strategies will enhance the specificity and sensitivity of the analysis of DNA methylation patterns over both CpG and non-CpG sites, and site-specific methylome editing will serve as a key technique for the study of 5-methylcytosine function, although both were still un-mature 5 years ago when the current study started. The percentage of monocytes, T cells and B cells in the PBMC samples was not determined, so we could not make corrections for cell-type composition in the best way^53^. However, the results open the possibility of using de-methylation in the treatment of ACO.

## Conclusions

We reported a novel association of ACO in adults of Asian origin with aberrant DNA methylation in the promoter or body regions of the *PDE9A, SEPT8,* and *ZNF323* genes. The findings extend reports linking hypomethylated *CYSLTR1/ADORA2B/CCDC88C* and hypermethylated *TIGIT* with more severe fixed airflow limitation in COPD patients, identifying hypomethylated *IFRD1*/hypermethylated *ZNF323* as biomarkers of frequent exacerbation, and providing direct evidence that perturbation of *MPV17L* signaling through epigenetic programming may play a role in the mediation of both inflammatory and allergic responses in ACO. Our findings provide a new direction for this disease and might establish novel biological insights into the development and effective treatment of ACO.

## Materials and methods

This study was approved by the Institutional Review Board of Chang Gung Memorial Hospital, Taiwan (certificate number: 103-3366B). The study participants were recruited from the pulmonary clinics of Kaohsiung Chang Gung Memorial Hospital from October 2014 to July 2017. All the participants were Taiwanese Han people in ancestry. Written informed consent was obtained from each subject participating in the study. The enrollment and exclusion criteria for COPD, and the definition of acute exacerbation (AE) were in accordance with GOLD guideline (Supplementary-Appendix 1 Text). ACO was defined by the presence of three elements: (I) COPD diagnosis, (II) positive bronchodilator (BD) test, and (III) blood eosinophil > 3%, or history of atopic diseases, including asthma, allergic rhinitis, or atopic dermatitis. A total of 364 subjects were screened, and 228 COPD patients were enrolled for final analysis. The discovery cohort used for the EWAS microarray experiment included 12 ACO patients and 6 healthy non-smokers (HS) with normal lung function. The non-overlapping validation cohort included 22 ACO patients, 48 patients with pure COPD, and 10 HS. The experiment, enrolment and exclusion criteria for COPD, and the definition of acute exacerbation (AE) were in accordance with GOLD guideline.

### DNA methylation measurement and analysis

Genome-wide DNA methylation profiles were measured by Infinium HumanMethylation 450 BeadChip v1.2 microarray method (San Diego, CA, USA). We filter out 485 CpG sites that have bead count smaller than 3 in 5% of total samples and filter out 901 CpG sites that have detection p-value greater than 0.01 in 5% of the total samples with R package wateRmelon^54^. We then transfer the methylation β value into M values^55^ which has better statistical properties for latter non-parametric statistical analysis. For those differentially methylated CpG sites, their corresponding gene symbols were used for pathway analysis and interaction networks contruction by MetaCore software (Thomson Reuters Incorporation, Philadelphia, USA). The significance threshold was a p < 0.0005 and a false discovery rate (q) < 0.3. All methylation datasets have been deposited in the NCBI Gene Expression Omnibus with the accession number GSE118468. Significantly differentially methylated CpG sites with at least a 10% difference in their β value (large effect size) and known biological or functional relevance were selected for further verification and validation by pyrosequencing method using PyroMark Q24 1.010 (Qiagen)^56^ (Supplementary Appendix 1 Text).

### In vitro human monocytic THP-1 cell culture under the stimuli with cigarette CSE and OVA allergen

THP-1 immortalized monocyte-like cell lines are derived from the peripheral blood of a childhood case of acute monocytic leukemia (M5 subtype) and represent valuable tools for investigating circulatory monocytes, which are one of the main sources of inflammatory cytokines in response to allergens and smoking exposures^57^. DNA Methyl-Transferase (DNMT) 3A and 3B mediate de novo deposition of C5-methylcytosine to establish methylation marks in CpG sites, while 5-aza is a chemical nucleoside analog of cytidine, which can incorporate into DNA, and trap DNMTs through an irreversible covalent interaction^58^. Thus, THP-1 and 5-aza were adopted in the in vitro experiments. THP-1 were treated with normal medium, 100 ng/ml lipopolysaccharide (LPS), 2.5% CSE, 25 ug OVA, or CSE (2.5%) plus OVA (25 ug) mix for 48 h, and also treated with 1uM 5-aza (Sigma-Aldrich Corp) in advance for 24 h. Gene expressions were measured by quantitative real-time reverse transcription-PCR method with Taqman probe and specific primers (Supplementary Table S7). Relative expression levels were calculated using the ∆∆Ct method. (Supplementary Appendix 1 Text).

### Statistical analysis

Data were expressed as the mean ± standard deviation. One-way analysis of variance were be used for comparing mean values of more than two experimental groups. Categorical variables were analyzed using Chi-square test. Stepwise multivariate linear regression analysis was used to adjust for confounding factors and obtain adjusted p values. The differences of continuous variables between study enrollment and follow-up after one year were analyzed by paired *t*-test. Pearson’s correlation was used to determine the relationship between selected variables. A p-value of less than 0.05 is considered statistically significant.

### Ethics approval and consent to participate

This study was approved by the Institutional Review Board of Chang Gung Memorial Hospital, Taiwan (certificate number: 103-3366B). Written informed consent was obtained from each subject participating in the study.

## Supplementary Information

Supplementary Information.

## Data Availability

All methylation datasets have been deposited in the NCBI Gene Expression Omnibus with the accession number GSE118468.

## Abbreviations

ACO: Asthma COPD overlap
ADORA2B: Adenosine A2b receptor
AE: Acute exacerbation
5-aza: 5-Aza-2′-deoxycytidine
BD: Bronchodilator
BMI: Body mass index
CCDC88C: Coiled-coil domain containing 88C
COPD: Chronic obstructive pulmonary disease
CpG: Cytosine guanine dinucleotides
CSE: Cigarette smoke extract
CYSLTR1: Cysteinyl leukotriene receptor 1
DML: Differentially methylated locus
EWAS: Epigenome-wide association study
FEV1: Forced expiratory volume in the first second
FVC: Forced expiratory vital capacity
HS: Healthy non-smoker
IFRD1: Interferon related developmental regulator 1
MPV17L: MPV17 mitochondrial inner membrane protein like
OVA: Ovalbumin
PCR: Polymerase chain reaction
PDE9A: Phosphodiesterase 9A
PTPRN2: Protein tyrosine phosphatase receptor type N2
SEPT8: Septin 8
SNP: Single nucleotide polymorphism
TIGIT: T cell immunoreceptor with Ig and ITIM domains
ZNF323: Zinc finger and SCAN domain containing 31
