# Supplementary material for: Epigenome-wide association study on asthma and chronic obstructive pulmonary disease overlap reveals aberrant DNA methylations related to clinical phenotypes
Source: Sci Rep. 2021 Mar 3;11:5022. doi: 10.1038/s41598-021-83185-1 (PMC7930096; doi:10.1038/s41598-021-83185-1)
Supplement: Supplementary file 1 — Supplementary Information. [file 41598_2021_83185_MOESM1_ESM.docx]

**Supplementary Appendix 1**

**Epigenome-wide association study on asthma and chronic obstructive**

**pulmonary disease overlap reveals aberrant DNA methylations related to**

**clinical phenotypes**

**Yung-Che Chen^a, b, #^, Ying-Huang Tsai^a^, Chin-Chou**

**Wang^a, f^, Shih-Feng Liu^a,g^, Ting-Wen Chen^c, d, e^, Wen-Feng Fang^a, f^, Chiu-Ping**

**Lee^a^, Po-Yuan Hsu^a^, Tung-Ying Chao^a^, Chao-Chien Wu^a^, Yu-Feng Wei^h^,**

**Huang-Chih Chang^a^, Chia-Cheng Tsen^a^, Yu-Ping Chang^a^, Meng-Chih Lin^a, b, #^, Taiwan Clinical Trial Consortium of Respiratory Disease (TCORE) group**

**Appendix 1 Text**

**The diagnostic criteria for COPD were as follows:** (I) male older than 40 years of age, (II) a smoking history of ≧10 pack-years, (III) post-bronchodilator (BD) forced expiratory volume in one-second (FEV1)/forced expiratory vital capacity (FVC)<70%. BD responsiveness was defined as having a post-BD improvement in FEV1 of > 200 ml and 12%. Those with a history of tuberculosis, bronchiectasis, malignant neoplasms, or recent admission (≦ 4 weeks) were excluded.

Acute exacerbation (AE) of COPD was defined as worsening of at least two major symptoms (dyspnea, sputum volume, and sputum purulence). Moderate AE was defined as prescription of oral antibiotics or systemic steroid, while severe AE as staying at emergency room for >24 hour or admission.

**Bisulfite conversion of genomic DNA**

Peripheral blood mononuclear cells (PBMCs) were isolated by Ficoll-Hypaque method, and genomic DNA were isolated from aliquots of PBMCs using Gentra Puregene Blood kit (Qiagen, Chatsworth, CA) following the manufacturer’s instructions. DNA samples for Infinium Methylation assay were bisulfite converted using EZ DNA methylation kit (Cat. #D5001) from Zymo Research (CA, USA). 500 ng of gDNA was denatured by addition of Zymo M-Dilution buffer and incubated for 15 min at 37 °C. CT-conversion reagent (bisulfite-containing) was added to the denatured DNA and incubated for 16 h at 50 °C in a thermocycler and denatured every 60 min by heating to 95 °C for 30 s. DNA samples for the whole-genome bisulfite sequencing were bisulfite converted using EpiTect Bisulfite conversion kit (Cat. #59104) from QIAGEN (Valencia, CA) following manufacturer's recommendations with modifications.

**Illumina’s Infinium DNA methylation assay**

As described previously, 4 μl of bisulfite-converted DNA were used for hybridization on Infinium HumanMethylation 450 BeadChip, following the Illumina Infinium HD Methylation protocol. BeadChip v1.2 (San Diego, CA, USA) was used to detect 482,421 methylation CpG sites genome-wide, located across gene regions with sites in the promoter region, 5'UTR, first exon, gene body, and 3'UTR of 14,495 genes, with the distance to the transcription start site ranging from 0 to 1499 bp[^1^](#_ENREF_1). The methylation score for each CpG was represented as a β value according to the fluorescent intensity ratio, and may take any value between 0 (non-methylated) and 1 (completely methylated). Methylation level of each CpG locus was calculated in GenomeStudio Methylation module as methylation beta-value (β= intensity of the Methylated allele (M)/(intensity of the Unmethylated allele (U)+intensity of the Methylated allele (M)+100). The raw β-value was then transformed into a M-value to achieve better statistical properties ^[2](#_ENREF_2)^, which was the log_2_ ratio of the intensity of methylated probes versus unmethylated probes using the following equation: M value = log_2_ (β value/(1-β value)). A positive M-value means higher intensity from the methylated probes than the un-methylated probes and a negative M value means the opposite. To identify differential methylated loci (DML), M values of the case and control groups were analyzed with the Mann-Whitney test by Partek Genomics Suite software (Louis, Missouri, U.S.A.) to obtain a p value and false discovery rate (q value).The significance threshold in M value comparisons was a p<0.005 and a q <0.5. All methylation datasets have been deposited in the NCBI Gene Expression Omnibus with the accession number GSE118468. The nearest genes for all the differentially methylated probes were identified for pathway enrichment analysis, which was conducted with hypergeometric test and the pathway databases built in Metacore (Thomson Reuters Incorporation, Philadelphia, USA).

**Measurement of CpG-site-specific DNA methylation levels by bisulfite pyro-sequencing method in the validation cohort**

A total of 24 CpG sites of 20 selected genes, including *PDE9A, SEPT8, IRFD1, CYSLTR1, TIGIT, ADORA2B, CCDC88C, NLRC5, CXCR5, TREX1, PRR5L, GFI1*, *DENND3*, *ZNF323*, *MPV17L*, *PTPRN2*, *PIK3CG*, *DUS2L*, *NBR2*, and *CTLA4*, were assayed. Several regions of the gene promoter or body element were amplified. Bisulfate treatment was performed using EpiTect 96 Bisulfite Kit (Qiagen) and PCR amplification was performed using PyroMark PCR Kit (Qiagen), as described previously[^3^](#_ENREF_3)^,^[^4^](#_ENREF_4). The PCR condition was 45 cycles of 95°C for 20 s, 50°C for 20 s, and 72°C for 20 s, followed by 72°C for 5 min. Primer sequences used for PCR amplification and pyro-sequencing for the 20 genes were listed in **supplementary-Table S1**. The biotin-labeled PCR product was captured by Streptavidin-Sepharose HP (Amersham Pharmacia). Quantitation of cytosine methylation was done using the PyroMark Q24 system (Qiagen). The amount of C relative to the sum of the amounts of C and T at each CpG site was calculated as percentage.

**In vitro human monocytic THP-1 cell culture under the stimuli with cigarette smoke extracts (CSE) and ovalbumin (OVA) allergen**

Davidoff cigarettes containing 10 mg of tar and 0.8 mg of nicotine per cigarette were used for preparing the smoke extracts by modifications of previous methods[^5^](#_ENREF_5)^,^[^6^](#_ENREF_6). Main stream smoke from twenty cigarettes was distributed via a manifold to capillaries submerged in a 50-ml centrifuge tube containing 15ml RPMI 1640 medium, which was then adjusted to pH 7.4 and filtered through a 0.22-μm filter. The concentration of CSE was calculated by measuring the optical density (OD) at a wavelength of 320 nm and this medium was defined as 100% CSE. Extracts were separated into aliquots and stored at -20°C. The amount of viable THP-1 cells under the stimuli with different concentration of CSE (1.25-10 %) or OVA (12.5-100 ug) for 24 h or 48 h was determined via OD measurement of WST-1 reagent (Roche, Mannheim, Germany) diluted at 1:10 using a microplate reader at 450 nm, with 600 nm as a reference wavelength. 0.1 μM of working solution of fluorochrome marker H2DCFDA (catalog no. D6883; Sigma, USA) was added to the THP-1 cells, which were submit to flow cytometry for reactive oxygen species (ROS) detection using the 488 nm laser for excitation and detected at 535 nm.

THP-1 were grown in a 12 well plate (10^5^ cells/well) for 24 h and then

treated with normal medium, 100 ng/ml lipopolysaccharide (LPS), 2.5% CSE, 25 ug OVA, or CSE (2.5 %) plus OVA (25 ug) mix for 48 h. DNA methylation levels and gene expressions of selected genes were measured by pyrosequencing and quantitative RT-PCR methods, as described above and below, respectively.

**Measurement of mRNA gene expressions by quantitative real-time reverse transcription (RT)-PCR method**

Total RNA from THP-1 cells was isolated by RNA Extraction RiboPureTM-Blood (Ambion), and converted to single-stranded cDNA using a cDNA archive kit (Applied Biosystems) followed by the amplification of the gene transcript by using Taqman probe and specific primers (**supplementary-Table S1**). *Glyceraldehyde-3-Phosphate Dehydrogenase* (*GAPDH*) was used as the internal control. The PCR reaction was performed at 94°C for 10 minutes, followed by amplification (95°C for 10 seconds, 60℃ for 30 seconds), and cooling (40°C for 30 seconds), for 30 cycles. The PCR products were subjected to 1% agarose gel electrophoresis and photographed. Relative expression levels were calculated using the ∆∆Ct method with the median value for the normal control (culture medium at 24 hours) group as the calibrator.

**References**

1 Chen, Y. C. *et al.* Whole Genome DNA Methylation Analysis of Obstructive Sleep Apnea: IL1R2, NPR2, AR, SP140 Methylation and Clinical Phenotype. *Sleep* **39**, 743-755, doi:10.5665/sleep.5620 (2016).

2 Du, P. *et al.* Comparison of Beta-value and M-value methods for quantifying methylation levels by microarray analysis. *BMC bioinformatics* **11**, 587, doi:10.1186/1471-2105-11-587 (2010).

3 Chen, Y. C. *et al.* Aberrant Toll-like receptor 2 promoter methylation in blood cells from patients with pulmonary tuberculosis. *The Journal of infection* **69**, 546-557, doi:10.1016/j.jinf.2014.08.014 (2014).

4 Chen, Y. C. *et al.* Increased S100A15 expression and decreased DNA methylation of its gene promoter are involved in high metastasis potential and poor outcome of lung adenocarcinoma. *Oncotarget* **8**, 45710-45724, doi:10.18632/oncotarget.17391 (2017).

5 Ostrow, K. L. *et al.* Cigarette smoke induces methylation of the tumor suppressor gene NISCH. *Epigenetics : official journal of the DNA Methylation Society* **8**, 383-388, doi:10.4161/epi.24195 (2013).

6 Rubenstein, D., Jesty, J. & Bluestein, D. Differences between mainstream and sidestream cigarette smoke extracts and nicotine in the activation of platelets under static and flow conditions. *Circulation* **109**, 78-83, doi:10.1161/01.CIR.0000108395.12766.25 (2004).

**Supplementary Figures**

**
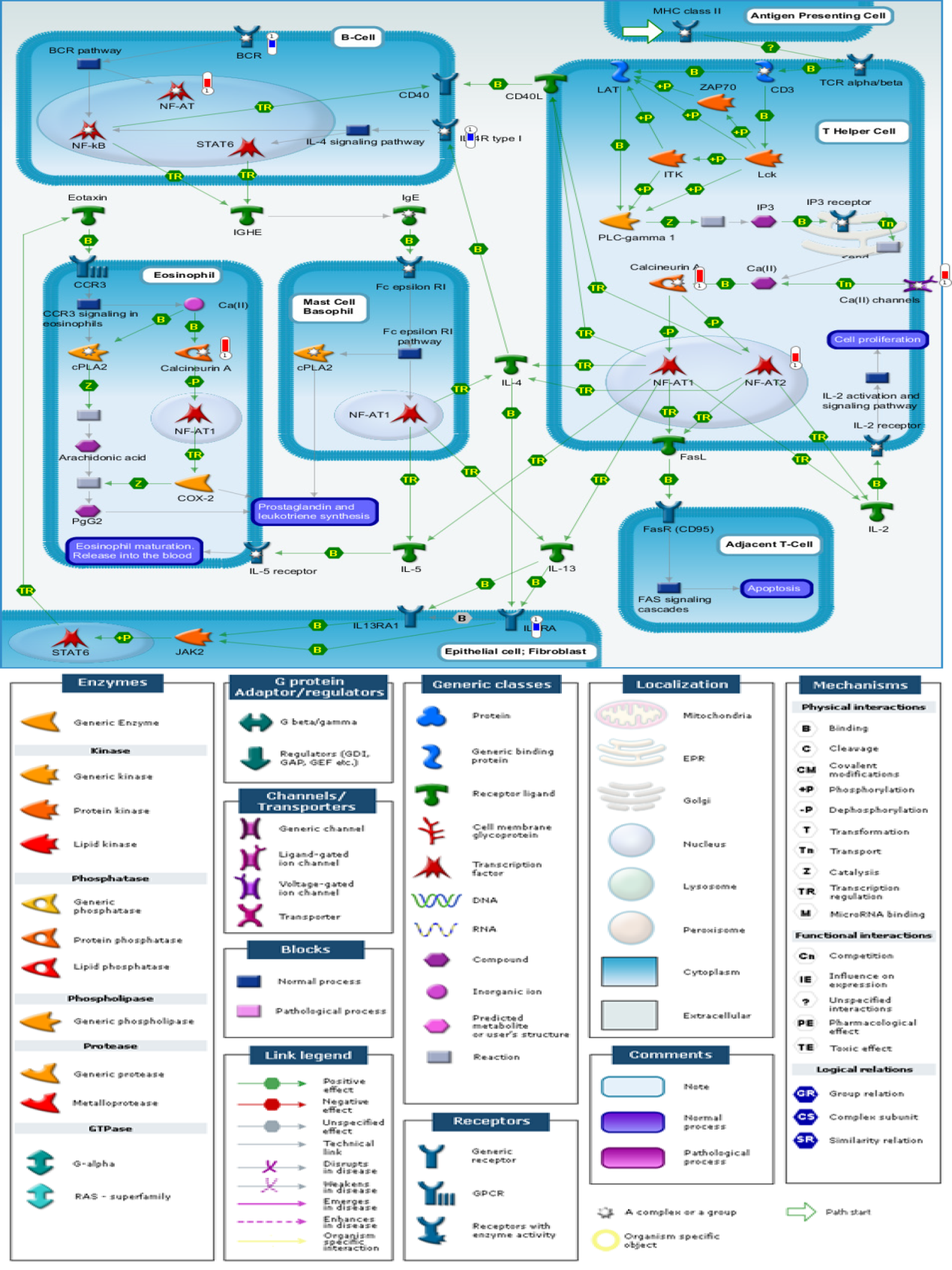
**

**Supplementary Figure 1. Immune response-NF AT signaling and leukocyte interactions signaling pathway enriched in ACO patients of the discovery cohort.** In comparison between ACO patients and healthy subjects, the significantly hypermethylated genes were highlighted with a red-colored barometric bar, while hypomethylated genes in a blue-colored bar. The changes represent the differences between the median M-values of normal and ACO. The image was created by the Metacore software.

**
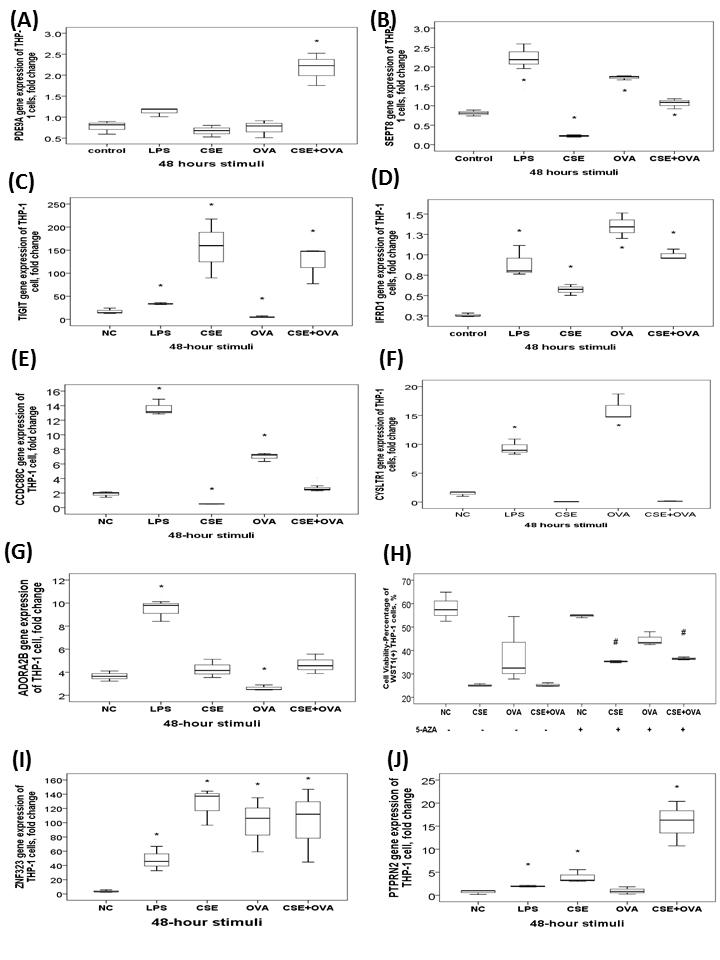
**

**Supplementary Figure 2. Seven candidate gene expressions in response to cigarette smoke extract (CSE) and ovalbumin (OVA) in vitro.** (A) PDE9A, (B) SEPT8, (C) TIGIT, and (D) IFRD1 gene expressions were all increased in response to CSE plus OVA treatment (all p values<0.05), while (E) CCDC88C and (F) CYSLTR1 gene expressions were both increased only in response to OVA stimuli (both p<0.05, supplementary Figure 1G and 1H). ADORA2B gene expression was decreased in response to OVA stimuli. (G) ZNF323 gene expression was increased in response to the stimuli with CSE, OVA, or CSE plus OVA (all p values<0.05). (H) PTPRN2 gene expression was increased in response to CSE or CSE plus OVA stimuli (P<0.05).

**Supplementary Table 1.** **Baseline characteristics of the participants in the DNA methylation microarray experiment (discovery cohort)**

|  | ACO,  N=12 | Healthy non-smokers, N=6 | P value |
| --- | --- | --- | --- |
| Age, years | 68.9 (9.5) | 64.3 (4.7) | 0.794 |
| Smoking exposure, pack-years | 54.7 (28) | 0 (0) | <0.001 |
| Current smoker | 6 (50) | 0 (0) | <0.001 |
| Body mass index, Kg/m2 | 24.2 (3.9) | 23.3 (3.2) | 0.66 |
| Charlson co-morbidity index | 2.7 (1.6) | 2.5 (1.6) | 0.916 |
| Atopic disease | 12 (100) | 0 (0) | <0.001 |
| Asthma | 5 (41.7) | 0 (0) | 0.063 |
| Allergic rhinitis | 11 (91.7) | 0 (0) | <0.001 |
| Atopic dermatitis | 0 (0) | 0 (0) |  |
| Lung function |  |  |  |
| Pre-BD FEV1/FVC, % | 58.7 (9.3) | 87.3 (5.7) | <0.001 |
| Pre-BD FEV1, %predicted | 58.7 (13.5) | 96.8 (8.7) | <0.001 |
| Pre-BD FEF25-75%, %predicted | 24.9 (10.3) | 103.2 (30.2) | <0.001 |
| Post-BD FEV1/FVC, % | 59.7 (10.3) | NA |  |
| Post-BD FEV1, %predicted | 63.2 (16.6) | NA |  |
| Post-BD FEF25-75%, %predicted | 29 (13.2) | NA |  |
| BD responsive | 12 (100) | NA |  |
| Dyspnea score |  |  |  |
| mMRC at the first visit | 2.4 (1.2) | 0.3 (0.5) | 0.001 |
| CAT at the first visit | 14.6 (7.3) | 1.7 (1) | 0.001 |
| Blood and biochemistry test |  |  |  |
| Neutrophil, % | 56.3 (14.6) | 57.2 (14) | 0.903 |
| Eosinophil, % | 9.1 (8.7) | 2.1 (0.7) | 0.017 |
| Absolute neutrophil count, μL^-1^ | 4391 (3728) | 3398 (1452) | 0.364 |
| Absolute eosinophil count, μL^-1^ | 900 (1569) | 114 (32) | 0.244 |
| Total cholesterol | 178.5 (23.8) | 169 (33.2) | 0.501 |
| Triglyceride | 107.5 (45.9) | 87.8 (29.6) | 0.367 |
| Uric acid | 7.6 (2.2) | 6.9 (1.7) | 0.519 |
| Glycohemoglobin | 5.8 (0.3) | 5.9 (0.8) | 0.762 |
| Exercise endurance test |  |  |  |
| Maximum inspiratory pressure, cmH2O | 71 (32.1) | NA |  |
| Maximum expiratory pressure, cmH2O | 90 (41.6) | NA |  |
| 6 minute walking distance, m | 424 (98) | NA |  |
| 6 minute walking distance, %predicted | 85.8 (17.1) | NA |  |

COPD= chronic obstructive pulmonary disease; ACO= asthma and COPD overlap; BD= bronchodilator, FEV1= forced expiratory volume within first second; FVC= forced expiratory vital capacity; FEF= forced expiratory flow; mMRC= modified Medical Research Council; CAT= COPD assessment test

**Supplementary Table 2. Top differentially methylated loci in the comparison (II) between asthma and COPD overlap (ACO) patients with rapid lung function decline after 1-year follow-up and at enrollment in the discovery cohort**

| Column ID | UCSC  RefGene  Name | UCSC  RefGene  Accession | UCSC  RefGene  Group | p-value | q-value | Meandifference of β value |
| --- | --- | --- | --- | --- | --- | --- |
| cg06361531 | *DUS2L* | NM_017803 | 5'UTR | 0.004396 | 0.299199 | 0.428 |
| ch.3.343413R | *NR2C2* | NM_003298 | Body | 0.004396 | 0.299199 | 0.42 |
| cg08460026 | *CTLA4* | NM_001037631 | TSS200 | 0.004396 | 0.299199 | 0.352 |
| cg08779777 | *PIK3CG* | NM_002649 | TSS200 | 0.004396 | 0.299199 | 0.323 |
| cg01035945 | *ZNF323* | NM_001135215 | 5'UTR | 0.004396 | 0.299199 | 0.298 |
| cg20604645 | *LOC100133161* | NR_028326 | TSS200 | 0.004396 | 0.299199 | 0.281 |
| cg20760063 | *NBR2* | NR_003108 | TSS200 | 0.004396 | 0.299199 | 0.279 |
| ch.3.55501R | *TRNT1* | NM_182916 | Body | 0.004396 | 0.299199 | 0.256 |
| cg07414487 | *PSORS1C1* | NM_014068 | Body | 0.004396 | 0.299199 | 0.254 |
| cg02399570 | *CCNL1* | NM_020307 | Body | 0.004396 | 0.299199 | 0.229 |
| cg21302120 | *DHX15* | NM_001358 | Body | 0.004396 | 0.299199 | 0.222 |
| cg01174743 | *ZDHHC14* | NM_153746630 | Body | 0.004396 | 0.299199 | 0.212 |
| cg21938894 | *TMEM151A* | NM_153266 | Body | 0.004396 | 0.299199 | 0.203 |
| cg10528424 | *SYT8* | NM_138567 | Body | 0.004396 | 0.299199 | 0.198 |
| cg27212541 | *DAG1* | NM_004393 | TSS200 | 0.004396 | 0.299199 | 0.193 |
| cg17466768 | *DNAJC27* | NM_016544 | Body | 0.004396 | 0.299199 | 0.189 |
| cg05655647 | *ATF1* | NM_005171 | TSS1500 | 0.004396 | 0.299199 | 0.176 |
| cg09844907 | *MPV17L* | NM_173803 | 1stExon | 0.004396 | 0.299199 | 0.164 |
| cg19165274 | *AKR1E2* | NM_001040177 | TSS200 | 0.004396 | 0.299199 | 0.163 |
| cg17479131 | *LOC401431* | NR_027040 | Body | 0.004396 | 0.299199 | 0.162 |
| cg04609859 | *HOXB4* | NM_024015 | 1stExon | 0.004396 | 0.299199 | 0.159 |
| cg06739680 | *LOC284551* | NR_027085 | TSS1500 | 0.004396 | 0.299199 | 0.158 |
| cg23554932 | *FAM66B* | NR_027423 | Body | 0.004396 | 0.299199 | 0.158 |
| cg23785719 | *STK17A* | NM_004760 | TSS200 | 0.004396 | 0.299199 | 0.155 |
| cg08215925 | *FOXP1* | NM_032682 | TSS200 | 0.004396 | 0.299199 | 0.153 |
| cg27119904 | *RASA3* | NM_007368 | Body | 0.004396 | 0.299199 | 0.148 |
| cg22407822 | *GNAS* | NM_001077490 | 3'UTR | 0.004396 | 0.299199 | 0.147 |
| cg15243856 | *MATN4* | NM_003833 | 5'UTR | 0.004396 | 0.299199 | 0.146 |
| cg07835289 | *LYST;LYST* | NM_000081 | 5'UTR | 0.004396 | 0.299199 | 0.143 |
| cg06442199 | *BRDT* | NM_207189 | TSS1500 | 0.004396 | 0.299199 | 0.142 |
| cg00466136 | *NTM* | NM_001048209 | Body | 0.004396 | 0.299199 | 0.142 |
| cg12065531 | *RING1* | NM_002931 | 1stExon | 0.004396 | 0.299199 | 0.141 |
| cg06009330 | *PCYOX1* | NM_016297 | 1stExon | 0.004396 | 0.299199 | 0.14 |
| cg06172626 | *TBC1D25* | NM_002536 | 1stExon | 0.004396 | 0.299199 | 0.139 |
| cg08409225 | *OBFC2B* | NM_024068 | TSS1500 | 0.004396 | 0.299199 | 0.135 |
| cg18793367 | *CUX1* | NM_181500 | Body; | 0.004396 | 0.299199 | 0.133 |
| cg13161025 | *LOC84856* | NR_026827 | Body | 0.004396 | 0.299199 | 0.132 |
| cg21604516 | *PURG* | NM_013357 | TSS200 | 0.004396 | 0.299199 | 0.131 |
| cg27226043 | *HCCA2* | NM_053005 | Body | 0.004396 | 0.299199 | 0.131 |
| cg23623667 | *KCNQ1* | NM_000218 | TSS1500 | 0.004396 | 0.299199 | 0.131 |
| cg25275011 | *ATP8B3* | NM_138813 | Body | 0.004396 | 0.299199 | 0.13 |
| cg13271751 | *CCND1* | NM_053056 | TSS1500 | 0.004396 | 0.299199 | -0.13 |
| cg10006408 | *WDR89* | NM_001008726 | TSS1500 | 0.004396 | 0.299199 | -0.132 |
| cg01702905 | *PCNX* | NM_014982 | TSS1500 | 0.004396 | 0.299199 | -0.14 |
| cg20476019 | *SYN3* | NM_133633 | Body | 0.004396 | 0.299199 | -0.14 |
| cg24421216 | *MEIS1* | NM_002398 | Body | 0.004396 | 0.299199 | -0.143 |
| cg06139006 | *GALNT9* | NM_001122636 | Body | 0.004396 | 0.299199 | -0.148 |
| cg02890259 | *HSPB7* | NM_014424 | 1stExon | 0.004396 | 0.299199 | -0.155 |
| cg24834873 | *ANKRD34B* | NM_001004441 | 5'UTR | 0.004396 | 0.299199 | -0.167 |
| cg04468334 | *KRBA1* | NM_032534 | 5'UTR | 0.004396 | 0.299199 | -0.171 |
| cg22033530 | *SYT5* | NM_003180 | TSS1500 | 0.004396 | 0.299199 | -0.181 |
| cg23015327 | *FAM101B* | NM_182705 | Body | 0.004396 | 0.299199 | -0.194 |
| cg25830305 | *TNNI2* | NM_003282 | TSS1500 | 0.004396 | 0.299199 | -0.197 |
| cg20550012 | *PTPRN2* | NM_002847 | Body | 0.004396 | 0.299199 | -0.291 |
| cg11897887 | *CST9L* | NM_080610 | TSS1500 | 0.004396 | 0.299199 | -0.358 |
| cg21795255 | *SLC2A9* | NM_020041 | Body | 0.004396 | 0.299199 | -0.505 |

COPD= chronic obstructive pulmonary disease; ACO= asthma and COPD overlap

**Supplementary Table 3. Nineteen differentially methylated loci overlapping between the comparison I and previous asthma-related DNA methylation probes**

| Column ID | UCSC  RefGene  Name | UCSC  RefGene  Accession | UCSC  RefGene  Group | Mean difference of β value | p-value | q-value |
| --- | --- | --- | --- | --- | --- | --- |
| cg16093065 | TNRC6B | NM_001162501 | 3'UTR | 0.179 | 0.000431 | 0.174101 |
| cg22116492 | MET | NM_001127500 | Body | 0.0779 | 0.000431 | 0.174101 |
| cg08711281 | DPCR1 | NM_080870 | TSS1500 | 0.0769 | 0.000431 | 0.174101 |
| cg20715809 |  |  |  | 0.0508 | 0.000431 | 0.174101 |
| cg10257332 | DHX30 | NM_138615 | Body | -0.0558 | 0.000431 | 0.174101 |
| cg15428620 | SFXN3 | NM_030971 | Body | -0.0596 | 0.000431 | 0.174101 |
| cg16243019 | DLX6AS | NR_015448 | Body | -0.0599 | 0.000215 | 0.08705 |
| cg03289906 | PPP1R9A | NM_001166161 | TSS1500 | -0.0665 | 0.000431 | 0.174101 |
| cg04783231 | ZFP28 | NM_020828 | Body | -0.0672 | 0.000215 | 0.08705 |
| cg05024939 |  |  |  | -0.0688 | 0.000215 | 0.08705 |
| cg25219643 | CCDC81 | NM_021827 | Body | -0.0755 | 0.000108 | 0.043525 |
| cg17879376 | BAI2 | NM_001703 | Body | -0.0868 | 0.000431 | 0.174101 |
| cg21846903 | VTN | NM_000638 | 1stExon | -0.0916 | 0.000215 | 0.08705 |
| cg25482146 | KDM2B | NM_032590 | Body | -0.0941 | 0.000431 | 0.174101 |
| cg07130150 |  |  |  | -0.0946 | 0.000108 | 0.043525 |
| cg06372718 | TRABD | NM_025204 | TSS1500 | -0.104 | 0.000215 | 0.08705 |
| cg01534527 | CTDSPL | NM_005808 | Body | -0.123 | 0.000215 | 0.08705 |
| cg08035555 | C19orf28 | NM_021731 | TSS1500 | -0.136 | 0.000431 | 0.174101 |
| cg05413628 | CLCN7 | NM_001287 | Body | -0.157 | 0.000431 | 0.174101 |

**Supplementary Table 4. Top 10 pathways enriched in the comparison between ACO patients and healthy non-smokers (comparison Ⅰ) in the discovery cohort**

| **Map** | **In Data/Total** | **p-**  **value** | **False Discovery Rate** | **Genes from Active Data** |
| --- | --- | --- | --- | --- |
| [Apoptosis and survival_APRIL and BAFF signaling](http://pathway.cgu.edu.tw/cgi/imagemap.cgi?id=2375) | 4/39 | 1.045E-03 | 2.879E-01 | *NF-AT2(NFATC1), Calcineurin A (catalytic), Bcl-2, CD21* |
| [Immune response_NF-AT signaling and leukocyte interactions](http://pathway.cgu.edu.tw/cgi/imagemap.cgi?id=2227) | 4/46 | 1.948E-03 | 2.879E-01 | *NF-AT2(NFATC1), Calcineurin A (catalytic), NF-AT, IL4RA* |
| [Development_Role of HDAC,calcium/calmodulin-dependent kinase (CaMK) in control of skeletal myogenesis](http://pathway.cgu.edu.tw/cgi/imagemap.cgi?id=440) | 4/54 | 3.517E-03 | 2.879E-01 | *HDAC4, L-type Ca(II) channel, alpha 1C subunit, Calcineurin A (catalytic), HDAC5* |
| [Development_G-CSF-induced myeloid differentiation](http://pathway.cgu.edu.tw/cgi/imagemap.cgi?id=6415) | 4/30 | 4.965E-03 | 2.879E-01 | *C/EBPalpha, GFI-1, PERM* |
| [Development_Regulation of epithelial-to-mesenchymal transition (EMT)](http://pathway.cgu.edu.tw/cgi/imagemap.cgi?id=3018) | 4/64 | 6.467E-03 | 2.879E-01 | *HGF receptor (Met), NOTCH4, FGFR1, Bcl-2* |
| [Cardiac Hypertrophy_NF-AT signaling in Cardiac Hypertrophy](http://pathway.cgu.edu.tw/cgi/imagemap.cgi?id=2235) | 4/65 | 6.831E-03 | 2.879E-01 | *GAB1, HDAC4, Calcineurin A (catalytic), HDAC5* |
| [Signal transduction_cAMP signaling](http://pathway.cgu.edu.tw/cgi/imagemap.cgi?id=660) | 3/38 | 9.646E-03 | 2.879E-01 | *KDELR, Calcineurin A (catalytic), cAMP-GEFI* |
| [Reproduction_GnRH signaling](http://pathway.cgu.edu.tw/cgi/imagemap.cgi?id=3137) | 4/72 | 9.758E-03 | 2.879E-01 | *PER1, HDAC4, L-type Ca(II) channel, alpha 1C subunit, HDAC5* |
| [Cell adhesion_PLAU signaling](http://pathway.cgu.edu.tw/cgi/imagemap.cgi?id=677) | 3/39 | 1.036E-02 | 2.879E-01 | *GAB1, HGF receptor (Met), Vitronectin* |
| [Development_VEGF-family signaling](http://pathway.cgu.edu.tw/cgi/imagemap.cgi?id=445) | 3/41 | 1.189E-02 | 2.879E-01 | *Neuropilin-1, Vitronectin, VEGF-A* |

COPD=chronic obstructive pulmonary disease; ACO= asthma and COPD overlap

**Supplementary Table 5.** **Top 10 pathways enriched in the comparison (II) between three asthma COPD overlap (ACO) patients with rapid lung function decline after 1-year medical treatment and at diagnosis in the discovery cohort.**

| Map | In Data/Total | P value | False Discovery Rate | Genes from Active Data |
| --- | --- | --- | --- | --- |
| [Cardiac Hypertrophy_NF-AT signaling in Cardiac Hypertrophy](http://pathway.cgu.edu.tw/cgi/imagemap.cgi?id=2235) | 44/65 | 4.937E-12 | 3.5247E-09 | HDAC9, gp130, ERK5 (MAPK7), Calcineurin A (catalytic), GRB2, GAB1, IRS-1, MEF2C, HAND1, NF-AT3(NFATC4), GATA-4, HDAC4, HDAC7, ADSSL1, G-protein alpha-q/11, beta-MHC, PKC-epsilon, Shc, GSK3 beta, PI3K reg class IA, ANP, MAP2K5 (MEK5), LIF, IL-6, IGF-1 receptor, ACTC, G-protein beta/gamma, AKT(PKB), c-Src, PI3K cat class IA, G-protein alpha-i family, HDAC5, HAND2, CSX (Nkx2.5), MEF2D, G-protein alpha-s, Calmodulin, PKC-alpha, SHP-2, NCX1, CaMKK, Beta-1 adrenergic receptor, Troponin T, cardiac, LIF receptor |
| [Development_Regulation of epithelial-to-mesenchymal transition (EMT)](http://pathway.cgu.edu.tw/cgi/imagemap.cgi?id=3018) | 42/64 | 7.044E-11 | 2.5148E-08 | VE-cadherin, TGF-beta 1, EGF, PDGF-B, NOTCH1 receptor, E2A, Lef-1, Sno-N, Caldesmon, TGIF, PDGF-R-alpha, Fibronectin, FGFR1, IL-1 beta, SIP1 (ZFHX1B), IL-1RI, NOTCH4, TWIST1, TNF-R1, PDGF-D, HGF, N-cadherin, E-cadherin, TGF-beta 2, TGF-beta receptor type II, EGFR, ZO-1, PDGF-A, TGF-beta 3, WNT, Bcl-2, Oncostatin M, Occludin, ACTA2, Tropomyosin-1, HGF receptor (Met), Jagged1, MMP-9, SRF, Frizzled, EDNRA, PDGF-R-beta |
| [Cytoskeleton remodeling_TGF, WNT and cytoskeletal remodeling](http://pathway.cgu.edu.tw/cgi/imagemap.cgi?id=715) | 60/111 | 7.361E-10 | 1.7519E-07 | Casein kinase II, alpha chains, Tuberin, TGF-beta 1, GRB2, TCF7L2 (TCF4), ERK1/2, Alpha-actinin, CRK, c-Raf-1, VEGFR-2, p21, Fibronectin, WIF1, Actin, Talin, Shc, GSK3 beta, PI3K reg class IA, PLAT (TPA), LRP5, CDC42, Actin cytoskeletal, LIMK2, ERK2 (MAPK1), AKT(PKB), Collagen IV, MYLK1, c-Src, ERK1 (MAPK3), Axin, MLCK, Casein kinase II, beta chain (Phosvitin), PI3K cat class IA, Tcf(Lef), TGF-beta receptor type II, XIAP, MEK3(MAP2K3), DOCK1, Laminin 1, SOS, Caspase-9, PAK1, SARA, PLAU (UPA), Paxillin, 4E-BP1, p130CAS, WNT, MRLC, FOXO3A, FAK1, Cyclin D1, SMAD3, Alpha-actinin 1, Caveolin-1, MELC, p38 MAPK, Frizzled, Cofilin, mTOR |
| [Cell adhesion_Cadherin-mediated cell adhesion](http://pathway.cgu.edu.tw/cgi/imagemap.cgi?id=2122) | 22/26 | 1.026E-09 | 1.832E-07 | VE-cadherin, P-cadherin, AF-6, PTPR-mu, Actin, PTPRF (LAR), Cortactin, CDC42, Actin cytoskeletal, Fyn, c-Src, N-cadherin, E-cadherin, Formin, DEP-1, Fer, M-cadherin, Alpha-catenin, SHP-2, FAK1, Plakoglobin, HGF receptor (Met) |
| [Neurophysiological process_Receptor-mediated axon growth repulsion](http://pathway.cgu.edu.tw/cgi/imagemap.cgi?id=527) | 31/45 | 3.761E-09 | 4.4761E-07 | CDK5, GRB7, Semaphorin 4D, Neuropilin-1, LARG, c-Raf-1, Ephrin-A receptor 2, Plexin A1, Cortactin, CDC42, Actin cytoskeletal, Fyn, Ephexin, LIMK2, c-Src, Pleiotrophin (OSF1), Tubulin (in microtubules), Tiam1, B-Raf, Plexin A2, EGFR, Fer, Ephrin-A receptors, CRMP2, PAK1, PDZ-RhoGEF, Semaphorin 3A, Plexin B1, Ephrin-A, Cofilin, Syndecan-3 |
| [Development_Ligand-independent activation of ESR1 and ESR2](http://pathway.cgu.edu.tw/cgi/imagemap.cgi?id=2210) | 31/45 | 3.761E-09 | 4.4761E-07 | EGF, GRB2, ESR1 (nuclear), IRS-1, Neuregulin 1, ERK1/2, ErbB3, PKA-reg (cAMP-dependent), c-Raf-1, ESR2, Shc, Adenylate cyclase type V, ErbB2, PI3K reg class IA, IGF-1 receptor, ERK2 (MAPK1), AKT(PKB), PDK (PDPK1), ERK1 (MAPK3), PI3K cat class IA, Galpha(s)-specific amine GPCRs, NCOA3 (pCIP/SRC3), EGFR, SOS, p90RSK1, NCOA2 (GRIP1/TIF2), G-protein alpha-s, Cyclin D1, NCOA1 (SRC1), Caveolin-1, TFF1 |
| [Cell adhesion_Chemokines and adhesion](http://pathway.cgu.edu.tw/cgi/imagemap.cgi?id=716) | 54/100 | 5.466E-09 | 5.5751E-07 | TRIO, GRB2, CD44, SERPINE2, Caveolin-2, CCL2, ERK1/2, Alpha-actinin, CRK, c-Raf-1, VEGFR-2, CCR1, Fibronectin, Actin, Talin, Shc, GSK3 beta, GRO-1, PI3K reg class IA, PLAT (TPA), G-protein beta/gamma, CDC42, Actin cytoskeletal, LIMK2, AKT(PKB), Collagen IV, c-Src, IL8RB, Drebrin, PI3K cat class IA, B-Raf, Tcf(Lef), G-protein alpha-i family, G-protein alpha-o, DOCK1, Laminin 1, SOS, PAK1, Thrombospondin 1, LAMA4, PLAU (UPA), Paxillin, p130CAS, PIPKI gamma, GCP2, PTEN, Zyxin, Rap1GAP1, FAK1, Alpha-actinin 1, Caveolin-1, CD47, Syndecan-2, Cofilin |
| [Transcription_Androgen Receptor nuclear signaling](http://pathway.cgu.edu.tw/cgi/imagemap.cgi?id=2202) | 30/45 | 2.202E-08 | 1.9655E-06 | TGF-beta 1, EGF, AKT1, GRB2, c-Raf-1, p21, Kallikrein 3 (PSA), VIL2 (ezrin), Shc, GSK3 beta, IL-6, IGF-1 receptor, N-CoR, ERK2 (MAPK1), PAK6, Hic-5/ARA55, Pyk2(FAK2), TGF-beta receptor type II, EGFR, SOS, NCOA2 (GRIP1/TIF2), RAD9, STAT3, WNT, SCAP, Cyclin D1, NCOA1 (SRC1), SMAD3, S5AR2, Frizzled |
| [Cytoskeleton remodeling_Cytoskeleton remodeling](http://pathway.cgu.edu.tw/cgi/imagemap.cgi?id=714) | 53/102 | 4.317E-08 | 3.0637E-06 | Casein kinase II, alpha chains, TRIO, TGF-beta 1, GRB2, ERK1/2, Alpha-actinin, CRK, c-Raf-1, VEGFR-2, p21, Fibronectin, Talin, Shc, GSK3 beta, PI3K reg class IA, PLAT (TPA), eIF4G2, CDC42, Actin cytoskeletal, LIMK2, ERK2 (MAPK1), Collagen IV, MYLK1, c-Src, MLCK, Casein kinase II, beta chain (Phosvitin), PI3K cat class IA, Tcf(Lef), TGF-beta receptor type II, XIAP, MEK3(MAP2K3), DOCK1, Laminin 1, SOS, eIF4G1/3, PAK1, eIF4G1, MyHC, PLAU (UPA), Paxillin, 4E-BP1, p130CAS, PIPKI gamma, PTEN, Zyxin, MRLC, FAK1, SMAD3, Alpha-actinin 1, Caveolin-1, MELC, p38 MAPK, Cofilin |
| [Cytoskeleton remodeling_Reverse signaling by ephrin B](http://pathway.cgu.edu.tw/cgi/imagemap.cgi?id=529) | 23/31 | 4.456E-08 | 3.0637E-06 | c-Raf-1, GSK3 beta, NCK2 (Grb4), G-protein beta/gamma, Actin cytoskeletal, c-Src, Axin, SDF-1, CXCR4, WaspIP, Tubulin (in microtubules), PINCH, RGS3, G-protein alpha-i family, SOS, PAK1, Paxillin, Tubulin alpha, Ephrin-B, PINCH-2, FAK1, Ephrin-B receptors, WIRE |

COPD= chronic obstructive pulmonary disease; ACO= asthma and COPD overlap

**Supplementary Table 6. Baseline characteristics of the participants in the DNA methylation pyro-sequencing experiments (validation cohort)**

|  | ACO,  N=22 | Pure  COPD, N=48 | | | P  value* | | Healthy non-smokers, n=10 | P value# |
| --- | --- | --- | --- | --- | --- | --- | --- | --- |
| Age, years | 70.1±8.2 | 68.7±10.9 | | | 1.0 | | 64.7±5.0 | 0.71 |
| Smoking exposure, pack-years | 44.5±25.6 | | 48.6±27.8 | | | 1.0 | 0.5±1.3 | <0.001 |
| Current smoker, n (%) | 6 (26.1) | | 26 (55.3) | | | 0.021 |  |  |
| Body mass index, Kg/m2 | 24.3±4.3 | | 24.7±4.3 | | | 1.0 | 24.7±2.0 | 1.0 |
| Charlson co-morbidity index | 2.8±1.8 | | 2.4±1.6 | | | 1.0 | 1.2±1.4 | 0.042 |
| Atopic disease, n (%) | 19 (82.6) | | 13 (27.1) | | | <0.001 |  |  |
| Asthma | 10 (43.5) | | 3 (6.3) | | | <0.001 |  |  |
| Allergic rhinitis | 16 (69.6) | | 12 (25) | | | <0.001 |  |  |
| Atopic dermatitis | 1 (4.3) | | 1 (2.1) | | | 0.589 |  |  |
| Lung function |  | |  | | |  |  |  |
| Pre-BD FEV1/FVC, % | 53.4±9.6 | | 61.7±15 | | | 0.043 | 83.2±7.4 | <0.001 |
| Pre-BD FEV1, %predicted | 50.9±15.3 | | 64.8±11.7 | | | 0.017 | 99±14.3 | <0.001 |
| Pre-BD FEF25-75%, %predicted | 19.5±8.4 | | 35.9±26.7 | | | 0.02 | 96.1±28.6 | <0.001 |
| Post-BD FEV1/FVC, % | 54.8±9.1 | | 62.7±15.5 | | | 0.069 | NA |  |
| Post-BD FEV1, %predicted | 54.4±9.1 | | 62.5±21.0 | | | 0.023 | NA |  |
| Post-BD FEF25-75%, %predicted | 22.0±7.3 | | 35.8±26.0 | | | 0.057 | NA |  |
| BD responsive, n (%) | 22 (100) | | 16 (33.3) | | | <0.001 | NA |  |
| Dyspnea score |  | |  | | |  |  |  |
| mMRC at the first visit | 1.8±1.2 | | 1.5±1.2 | | | 1.0 | 0.4 (0.5) | 0.01 |
| CAT at the first visit | 11.7±7.6 | | | 7.9±6.1 | | 0.07 | 2.7 (2.8) | 0.001 |
| Blood and biochemistry test |  | | |  | |  |  |  |
| Neutrophil, % | 58.7±11.0 | | | 61.5±12.6 | | 1.0 | 55.0 (6.4) | 1.0 |
| Eosinophil, % | 5.8±4.8 | | | 2.9±1.9 | | <0.001 | 2.0 (1.4) | 0.007 |
| Absolute neutrophil count, μL^-1^ | 4600±1910 | | | 438±2049 | | 1.0 | 3220 (803) | 0.218 |
| Absolute eosinophil count, μL^-1^ | 398.7±333.1 | | | 166±101.8 | | <0.001 | 112.9 (79.2) | 0.001 |
| Total cholesterol | 182.5±35.7 | | | 186.8± 33.0 | | 1.0 | 169.0 ±19.4 | 0.902 |
| Triglyceride | 111.5 (43.3) | | 118.0 ±71.1 | | | 1.0 | 98.6 ±57.6 | 1.0 |
| Uric acid | 7.7±2.2 | | 6.7±1.8 | | | 0.16 | 5.8±1.6 | 0.037 |
| Glycohemoglobin | 5.9±0.5 | | 6.1±0.9 | | | 1.0 | 6.1±0.6 | 1.0 |
| Controller Medicines, n (%) |  | |  | | |  |  |  |
| LAMA | 13 (40.7) | | 20 (46.2) | | | 0.663 |  |  |
| LABA | 16 (51.9) | | 14 (30.8) | | | 0.085 |  |  |
| ICS+LABA | 17 (55.6) | | 11 (23.1) | | | 0.007 |  |  |
| Theophylline | 20 (66.7) | | 20 (46.2) | | | 0.1 |  |  |
| Exercise endurance test |  | |  | | |  |  |  |
| Maximum inspiratory pressure, cmH2O | 68 (30.7) | | 69.6 (33.5) | | | 0.869 | NA |  |
| Maximum expiratory pressure, cmH2O | 96 (33.6) | | 95.8 (33.3) | | | 0.984 | NA |  |
| 6 minute walking distance, m | 357(114.3) | | 381.6(127.6) | | | 0.488 | NA |  |
| 6 minute walking distance, %predicted | 74.3 (22) | | 82.6 (26.7) | | | 0.252 | NA |  |

*p value for comparisons between ACO and pure COPD patients

#p value for comparisons between ACO patients and healthy non-smokers (HS)

COPD= chronic obstructive pulmonary disease; ACO= asthma and COPD overlap; BD= bronchodilator, FEV1= forced expiratory volume within first second; FVC= forced expiratory vital capacity; FEF= forced expiratory flow; LAMA= long acting muscarinic antagonist; LABA= long acting β2 agonist; ICS= inhaled corticosteroid; mMRC= modified Medical Research Council; CAT= COPD assessment test

**Supplementary Table 7. Primer sequence used for polymerase chain reaction and pyro-sequencing of the six selected genes verified in the validation cohort**

| Gene name /  probe ID/  NCBI Reference | CpG location relative to transcription start site | Primer | Sequences |
| --- | --- | --- | --- |
| *PDE9A*  cg24450112  NM_001001582 | +30088 | Forward PCR Primer | agggtgagccacaagaaaggccag |
|  |  | Biotinylated Reverse PCR Primer | ctggcttgaagggggtgcacctaccagca |
|  |  | Forward Sequencing Primer | ctgtcccagcccctcagagtgc |
| *SEPT8*  cg13334727  NM_001098813 | -40/-47/-50 | Forward PCR Primer | gcagtttaggctgggattcctgcaatagaa |
|  |  | Biotinylated Reverse PCR Primer | gcctgtcccaagggcttgggac |
|  |  | Forward Sequencing Primer | gggattcctgcaatagaaaag |
| *TIGIT*  cg19421218  NM_173799 | -173 | Forward PCR Primer | agggtgagccacaagaaaggccag |
|  |  | Biotinylated Reverse PCR Primer | gagctcaagggcatctgtgtagtgc |
|  |  | Forward Sequencing Primer | attgtggttagaggtacac |
| *CYSLTR1*  cg00813999  NM_006639 | +348 | Forward PCR Primer | aggattaattgcaaggactggtagat |
|  |  | Biotinylated Reverse PCR Primer | agttagcaggtaaggagact  cagtat |
|  |  | Forward Sequencing Primer | ggagcttgcttctgagaa |
| *IFRD1*  cg10288111  [NM_001007245.2](https://www.ncbi.nlm.nih.gov/projects/sviewer/sequence.cgi?id=gi\|308193306&format=fasta&filename=NM_001007245.2.fa&ranges=0-3513) | -515 | Forward PCR Primer | gagggaggaaaaagcaagtaaaagatctg |
|  |  | Biotinylated Reverse PCR Primer | tagccagagttgtaaaaatggcttgc |
|  |  | Forward Sequencing Primer | gtgttttccaaaagatagatgga |
| *CCDC88C*  cg26290716  NM_001080414 | +125722/  +125731/  +125735 | Biotinylated Forward PCR Primer | gaaagctgcacttgtgggtgatg |
|  |  | Reverse PCR Primer | gacagaaaggagagaaatggatttc |
|  |  | Forward Sequencing Primer | gaatgaaggttggaaacac |
| *ADORA2B*  cg07563400  NM_000676 | +1326 | Forward PCR Primer | ggcccatcccaggggaaggtg |
|  |  | Biotinylated Reverse PCR Primer | gctgagaagtgcagaaaatggtgg |
|  |  | Forward Sequencing Primer | aaggagttacaatgcttcct |
| *MPV17L*  cg09844907  NM_001128423.1 | +194 | Forward PCR Primer | gggtccgggcctccccagcctccagcag |
|  |  | Biotinylated Reverse PCR Primer | agaacaggcaaagccacagaggcagaagg |
|  |  | Forward Sequencing Primer | tgcatctgccccctccagaagctc |
|  | -113 | Forward PCR Primer | ggaggttgaggtaggagaa |
|  |  | Biotinylated Reverse PCR Primer | cacccccatacataaatccat |
|  |  | Forward Sequencing Primer | gttgaggtaggagaat |
| *ZNF323;ZKSCAN3*  cg01035945  NM_001242894.1 | -296 | Forward PCR Primer | gggagaaaggcaaggtgcagaat |
|  |  | Biotinylated Reverse PCR Primer | agtgagaaggaaagtgttagggtg |
|  |  | Forward Sequencing Primer | ggaaaattaataatggtggtt |
| *PIK3CG* cg08779777  NM_001282426.1 | +49 | Forward PCR Primer | ctggatatgaagggagccccagaaa |
|  |  | Biotinylated Reverse PCR Primer | ctttgtggggtctgactcggaatag  tgg |
|  |  | Forward Sequencing Primer | gaagggagccccagaaaag |
| *NBR2*  cg20760063 NR_138145.1 | +6 | Biotinylated Forward PCR Primer | gggctctggattggccacccagt |
|  |  | Reverse PCR Primer | ggtaaatataagtaataaggattgttgggg |
|  |  | Forward Sequencing Primer | gaggaagaattctacctgagt |
| *CTLA4*  cg08460026  NM_005214.4 | -36 | Forward PCR Primer | gtgttcaggtcttcaggaagtagagca |
|  |  | Biotinylated Reverse PCR Primer | ctaagtggagacttggagaatttcc |
|  |  | Forward Sequencing Primer | agaaagcctttttgttttgg |
| *DUS2L;DDX28*  cg06361531 NM_018380.3 | -10 | Forward PCR Primer | gtttctgacggggaagccagtggact |
|  |  | Biotinylated Reverse PCR Primer | gagtgaaccttggcttaaaaggaaggaa |
|  |  | Forward Sequencing Primer | gggttctggatgagaa |
| PTPRN2  cg20550012  NM_130842.3 | +10000/  +10007/  +10015 | Biotinylated Forward PCR Primer | agagccacccagggcaggacccaccagc |
|  |  | Reverse PCR Primer | aaccagaccctgggagtgtggtg |
|  |  | Forward Sequencing Primer | gtggatggttcacacggctgt |
| *TREX1*  *cg01870865*  NM_033629.5 | -263 | Forward PCR Primer | caacagccagggacaggaagaaggatccca |
|  |  | Biotinylated Reverse PCR Primer | gaaacaaggccagccagttctctgggc |
|  |  | Forward Sequencing Primer | agatcagactcaggaaacaaggc |
| *CXCR5*  *cg19791714*  NM_001716.4 | +9325 | Forward PCR Primer | gaggagggagcccacaggccaagtca |
|  |  | Biotinylated Reverse PCR Primer | tcagaactggaacagatgggtccttgt |
|  |  | Forward Sequencing Primer | agcacaatgctaagttgcagtg |
| *NLRC5*  *cg05757530* NM_001330552.1 | +15519 | Forward PCR Primer | aaggtggagggaaaatggccag |
|  |  | Biotinylated Reverse PCR Primer | gggctggggtgcacctggaagattta |
|  |  | Forward Sequencing Primer | gggaaaatggccagggtca |
| *PRR5L*  *cg26601310*  NM_001160167.1 | +79398 | Biotinylated Forward PCR Primer | tcttgctcagttttgagataaaaggacatg |
|  |  | Reverse PCR Primer | ccatagggtgaaatgatgcaggtagtg |
|  |  | Forward Sequencing Primer | gggcagcatttttaaagtatttcct |
| *GFI1*  *cg07805029*  NM_005263.4 | **-**884 | Forward PCR Primer | tttgcagggaatatttggttagtatagc |
|  |  | Biotinylated Reverse PCR Primer | aggcaagcaattttggtcagaaagga |
|  |  | Forward Sequencing Primer | atttggttagtatagcagtgc |
| DENND3  cg11307715  [XR_928310.3](https://www.ncbi.nlm.nih.gov/projects/sviewer/sequence.cgi?id=gi\|1370512104&format=fasta&filename=XR_928310.3.fa&ranges=0-5635) | **+**23226 | Biotinylated Forward PCR Primer | tggttattgagtgttttatggtttat |
|  |  | Reverse PCR Primer | actcacaaaaatttaacattccaaatca |
|  |  | Forward Sequencing Primer | cctaaccttactaacttctt |

PCR= polymerase chain reaction; CpG= cytosine guanine dinucleotide
